# Molecular and physiological changes in the SpaceX Inspiration4 civilian crew

**DOI:** 10.1038/s41586-024-07648-x

**Published:** 2024-06-11

**Authors:** Christopher W. Jones, Eliah G. Overbey, Jerome Lacombe, Adrian J. Ecker, Cem Meydan, Krista Ryon, Braden Tierney, Namita Damle, Matthew MacKay, Evan E. Afshin, Jonathan Foox, Jiwoon Park, Theodore M. Nelson, Mir Suhail Mohamad, Syed Gufran Ahmad Byhaqui, Burhan Aslam, Ummer Akbar Tali, Liaqun Nisa, Priya V. Menon, Chintan O. Patel, Sharib A. Khan, Doug J. Ebert, Aaron Everson, Michael C. Schubert, Nabila N. Ali, Mallika S. Sarma, JangKeun Kim, Nadia Houerbi, Kirill Grigorev, J. Sebastian Garcia Medina, Alexander J. Summers, Jian Gu, John A. Altin, Ali Fattahi, Mohammad I. Hirzallah, Jimmy H. Wu, Alexander C. Stahn, Afshin Beheshti, Remi Klotz, Veronica Ortiz, Min Yu, Laura Patras, Irina Matei, David Lyden, Ari Melnick, Neil Banerjee, Sean Mullane, Ashley S. Kleinman, Michael Loesche, Anil S. Menon, Dorit B. Donoviel, Emmanuel Urquieta, Jaime Mateus, Ashot E. Sargsyan, Mark Shelhamer, Frederic Zenhausern, Eric M. Bershad, Mathias Basner, Christopher E. Mason

**Affiliations:** 1 Unit for Experimental Psychiatry, Division of Sleep and Chronobiology, Department of Psychiatry, University of Pennsylvania Perelman School of Medicine, Philadelphia, PA USA; 2 Department of Physiology, Biophysics and Medicine, Weill Cornell Medicine, New York, NY USA; 3 The HRH Prince Alwaleed Bin Talal Bin Abdulaziz Alsaud Institute for Computational Biomedicine, Weill Cornell Medicine, New York, NY USA; 4 The WorldQuant Initiative for Quantitative Prediction, Weill Cornell Medicine, New York, NY USA; 5 Center for STEM, University of Austin, Austin, TX USA; 6 Center for Applied Nanobioscience and Medicine, College of Medicine-Phoenix, University of Arizona, Phoenix, AZ USA; 7 Department of Basic Medical Sciences, College of Medicine Phoenix, University of Arizona, Phoenix, AZ USA; 8 Department of Microbiology & Immunology, Vagelos College of Physicians & Surgeons, Columbia University Irving Medical Center, New York, NY USA; 9 TrialX Inc., New York, NY USA; 10 KBR, Science & Space, Houston, TX USA; 11 Department of Otolaryngology - Head & Neck Surgery, The Johns Hopkins University School of Medicine, Baltimore, MD USA; 12 The Translational Genomics Research Institute (TGen), Phoenix, AZ USA; 13 Departments of Neurology and Neurosurgery, Baylor College of Medicine, Houston, TX USA; 14 Center for Space Medicine, Baylor College of Medicine, Houston, TX USA; 15 The Translational Research Institute for Space Health (TRISH), Houston, TX USA; 16 Stanley Center for Psychiatric Research, Broad Institute of MIT and Harvard, Cambridge, MA USA; 17 Blue Marble Space Institute of Science, Space Biosciences Division, NASA Ames Research Center, Moffett Field, CA USA; 18 Department of Stem Cell Biology and Regenerative Medicine, Keck School of Medicine, University of Southern California, Los Angeles, CA USA; 19 Children’s Cancer and Blood Foundation Laboratories, Departments of Pediatrics and Cell and Developmental Biology, Drukier Institute for Children’s Health, Weill Cornell Medicine, New York, NY USA; 20 Department of Molecular Biology and Biotechnology, Center of Systems Biology, Biodiversity and Bioresources, Faculty of Biology and Geology, Babes-Bolyai University, Cluj-Napoca, Romania; 21 Meyer Cancer Center, Weill Cornell Medicine, New York, NY USA; 22 SpaceX, Hawthorne, CA USA; 23 University of Texas, Department of Emergency Medicine, Houston, TX USA; 24 Department of Biomedical Engineering, University of Arizona, Tucson, AZ USA

**Keywords:** Physiology, Molecular biology, Cognitive neuroscience, Oculomotor system, Visual system

## Abstract

Human spaceflight has historically been managed by government agencies, such as in the NASA Twins Study^1^, but new commercial spaceflight opportunities have opened spaceflight to a broader population. In 2021, the SpaceX Inspiration4 mission launched the first all-civilian crew to low Earth orbit, which included the youngest American astronaut (aged 29), new in-flight experimental technologies (handheld ultrasound imaging, smartwatch wearables and immune profiling), ocular alignment measurements and new protocols for in-depth, multi-omic molecular and cellular profiling. Here we report the primary findings from the 3-day spaceflight mission, which induced a broad range of physiological and stress responses, neurovestibular changes indexed by ocular misalignment, and altered neurocognitive functioning, some of which match those of long-term spaceflight^2^, but almost all of which did not differ from baseline (pre-flight) after return to Earth. Overall, these preliminary civilian spaceflight data suggest that short-duration missions do not pose a significant health risk, and moreover present a rich opportunity to measure the earliest phases of adaptation to spaceflight in the human body at anatomical, cellular, physiological and cognitive levels. Finally, these methods and results lay the foundation for an open, rapidly expanding biomedical database for astronauts^3^, which can inform countermeasure development for both private and government-sponsored space missions.

## Abstract (web summary)

SpaceX’s Inspiration4 mission sent an all-civilian crew into orbit to study physiological, neurovestibular and neurocognitive changes in the astronauts and found that short-duration civilian space missions do not pose a major health risk.

## Main

Orbital human spaceflight missions have historically flown highly screened and extensively trained cohorts of astronauts, with limited public data available for follow-up analyses. However, with the emergence of private space programmes and broadening access to orbital missions, new opportunities for research and discovery have emerged for civilian engagement with spaceflight. Specifically, SpaceX has now launched or announced several privately supported missions, including the all-civilian Inspiration4 mission and the Polaris Dawn series of missions (including on Starship). These missions enable a broader representation of astronaut cohorts, spanning a wider range of ages (for example, the youngest American astronaut, Hayley Arceneaux), balanced representation of sexes, diverse genetic and medical backgrounds, as well as opportunities for new science, technology, art and public engagement during their missions.

Here we present findings from the human research experiments performed on SpaceX’s first all-civilian private spaceflight, Inspiration4, which launched from Kennedy Space Center on 15 September 2021, on the SpaceX *Crew Dragon* capsule. This orbital class mission reached 590.6 km in altitude; the farthest distance crewed orbital missions have been into space since the Gemini programme. Although the crew spent only 3 days in low Earth orbit, they experienced similar hazards of spaceflight as International Space Station (ISS) missions (often 6–12 months in duration), including radiation exposure, sustained microgravity, the closed and hostile environment of space, isolation and confinement, and long distance from Earth resources^2^. Thus, the Inspiration4 mission, and similar missions, provide a platform to study these acute exposures endemic to low Earth orbit, with unique data collection opportunities in the earliest phase of the human body’s response to spaceflight.

**Extended Data Fig. 1:**
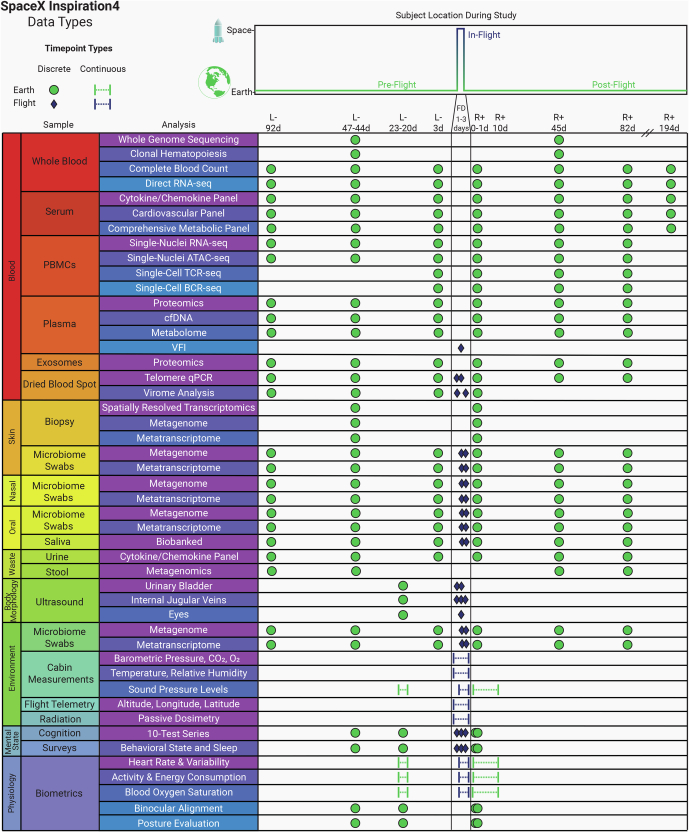
The environmental, physiological, cognitive, imaging, and omics measures collected across Inspiration4 mission phases and their sampling frequency. To assess the effect of short-duration spaceflight on an all-civilian crew, data were collected on myriad domains using various methodologies (colors, left) before, during, and after flight spanning human biometrics, virome, cognition, spacecraft environment, and multi-omics assays. Green circles denote samples collected on Earth (i.e., pre-flight, post-flight) and blue diamonds denote data collected in-flight, along with their respective assays. For the timing of data collection, data collected during the pre-flight period is presented as the number of days prior to launch (L) and data collected in the post-flight period is presented as the number of days following return (R) to Earth. Data with continual collection (e.g. cabin measurements) are shown as bracketed time periods.

The three main objectives of the research projects deployed on the Inspiration4 mission were to (1) evaluate the feasibility of collecting biological and behavioural data in an all-civilian crew throughout pre-flight, in-flight and post-flight phases of the spaceflight mission, (2) examine the biological and behavioural responses of the crew to short-duration orbital spaceflight and (3) build the foundation for a biomedical database and enable access to these biomedical data from the crew and mission. The Inspiration4 mission provided a unique opportunity to test the feasibility of medical research aboard a commercial spaceflight mission crewed by non-professional astronauts, as well as new clinical and research protocols, which spanned a wide range of assays and experiments (Extended Data Fig. 1 and Supplementary Table 1). Specifically, the Multimodal Evaluation of Spaceflight Health protocol for the Inspiration4 mission from the Translational Research Institute for Space Health (TRISH) funded multiple investigators to conduct separate yet related investigations on the Inspiration4 mission, and deployed a battery of tests to understand some of the major effects of short-duration spaceflight on humans, including portable ultrasound measurements, cognitive and sensorimotor tests, surveys, physiological data collected with a smartwatch and blood/saliva testing. Moreover, a comprehensive Space Omics and Medical Atlas (SOMA) protocol, based on the NASA Twins Study^1^, was used to measure multi-omic, clinical and immune profiles in whole blood, serum, plasma, saliva and biopsied skin samples. In this Article, we present and discuss the key findings from studies evaluating the Inspiration4 crew’s comprehensive multi-omics analyses of biospecimens, ultrasound imaging, otolith function, cardiovascular physiology and cognitive performance, which together enable an in-depth biomedical research approach for private crews on upcoming missions and unprecedented access to these data and samples.

## Multi-omic profiling of the Inspiration4 crew

**Fig. 1:**
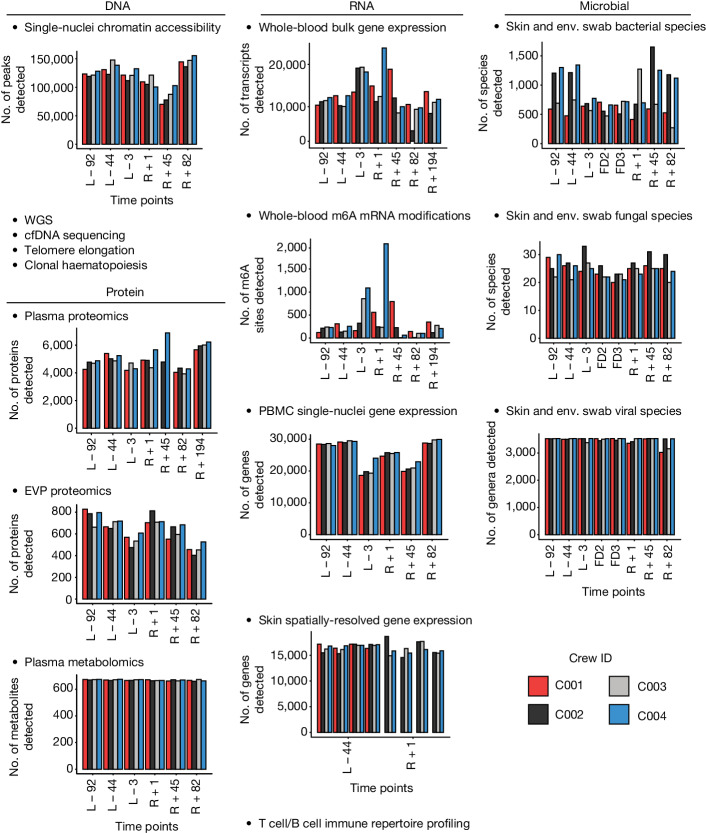
Multi-omic changes across the Inspiration4 mission. Hundreds of thousands of multi-omic measurements were generated across multiple sample types. From DNA, single-nuclei chromatin accessibility, WGS, cell-free DNA (cfDNA) sequencing, telomere length and clonal haematopoiesis were measured. From RNA, whole blood gene expression, whole blood m6A modifications, peripheral blood mononuclear cell (PBMC) single-nuclei RNA sequencing, skin spatially resolved gene expression and T cell and B cell immune repertoire profiling were performed. For proteins, plasma proteomics, extracellular vesicles and particles (EVP) proteomics and plasma metabolomics were quantified. Additionally, from microbial skin and environmental (Env.) swabs, bacterial, fungal and viral species were measured.

**Extended Data Fig. 2:**
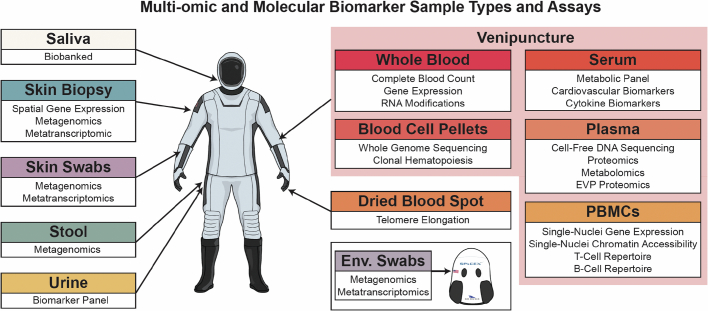
SOMA biosample collections during the Inspiration4 mission. Sample types and downstream assays performed for multi-omic and clinical biomarkers. A variety of blood derivatives were collected via venipuncture. Samples that have not yet been sequenced are biobanked. EVP = extracellular vesicles and particles. Env = environment. Created with BioRender.com.

Building upon established omics, sample pipelines and analytic methods from the NASA Twins Study^1^, we collected samples for an integrative multi-omics analysis of the Inspiration4 crew (Extended Data Fig. 2). This included biospecimens collected before launch (L) pre-flight (L − 92, L − 44 and L − 3), in-flight (flight days (FDs) FD1, FD2 and FD3) and post-flight following return (R) to Earth (R + 1, R + 45, R + 82 and R + 194). Blood, saliva, skin swabs, skin biopsies and capsule swabs were collected (Extended Data Fig. 2), followed by a battery of assays to collect multi-omic measurements, including spatially resolved transcriptomics, whole-genome sequencing (WGS), direct RNA sequencing, combined single-nuclei RNA sequencing/single-nuclei assay for transposase-accessible chromatin using sequencing (ATAC-seq), T cell receptor immune repertoire sequencing, B cell receptor immune repertoire sequencing, proteomics (liquid chromatography with tandem mass spectrometry (LC–MS/MS)), metabolomics (LC–MS/MS), a clonal haematopoiesis panel, cell-free DNA and cell-free RNA sequencing from plasma, shotgun metagenomics and shotgun metatranscriptomics (Methods and Extended Data Fig. 2). Also, clinical measurements were obtained and included complete blood count, a comprehensive metabolic panel, a CLIA-grade (Clinical Laboratory Improvement Amendments) WGS and pharmacogenomics profile, a cardiovascular biomarker panel and cytokine/chemokine panel assays (Fig. 1, Extended Data Fig. 2 and Supplementary Table 2).

These data revealed a broad set of molecular changes across multiple layers of biology (Fig. 1), and each specific omics dataset was examined in depth across several Inspiration4 companion papers. First, epigenetics data from single-nuclei chromatin (single-nuclei ATAC-seq) profiling showed more than 70,000 peaks per sample, which were enriched for genes involved in DNA repair, immune activation and nucleosome organization^4,5^, notably matching results from the NASA Twins Study^1^. Second, telomere elongation was observed for all crew members, but all other metrics of genome stability, sequence divergence and clonal haematopoiesis were unchanged^6^. Third, we observed an average of 668 metabolites and more than 4,000 proteins per sample in plasma, which was smaller than the 637 proteins per sample in the exosome population, yet the exosome proteomic data revealed unique, brain-associated peptides^7^. Fourth, direct RNA nanopore sequencing showed a mean of 13,022 transcripts per sample, plus a significant spike in methyl-6-adenine (m6A) levels on the day of landing, R + 1 (ref. ^8^); this was complemented with a range of 18,632–29,900 genes detected across all single-nucleus RNA sequencing data. Fifth, we characterized the spatial biopsies of the crew samples with GeoMx, which revealed a mean of 16,433 genes per section, with evidence of disrupted inflammation in pathways near the surface of the skin^9^. Finally, we examined the microbiome of the crew, using shotgun metagenomics and metatranscriptomics before, during and after spaceflight, and we found a spike in virus abundance in-flight, as well as 3.6 million non-redundant genes at 90% identity, 1,287 metagenomic assembled genomes and 1,544 assembled viral genomes^10^.

## Immune reactivity analysis

**Fig. 2:**
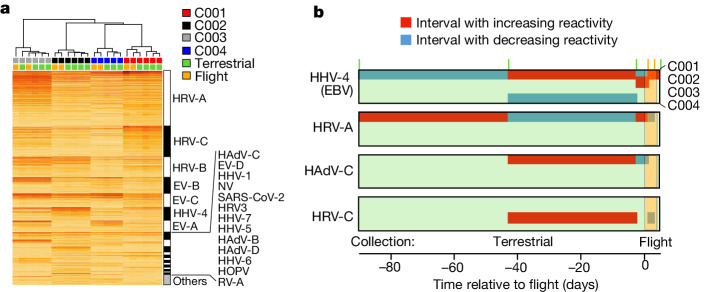
Virome-wide antibody analysis of blood samples self-collected during short-duration spaceflight. Blood spot samples were collected longitudinally across all mission phases from the four Inspiration4 astronauts and analysed for IgG reactivity across a 15,000-plex peptide library representing 80 human-infecting viral species using the PepSeq assay. **a**, Reactivity for each sample (columns) is shown across all 1,490 peptides (rows) reactive in at least one astronaut, with samples clustered by the similarity of their reactivity profiles and peptides grouped by the virus species from which they were designed. Sample reactivity profiles clustered tightly within each astronaut, including those collected in-flight. Reactivity was detected against peptides from a total of 45 virus species, listed in Supplemental Table 3, including full names. **b**, Time intervals with significantly increasing (red) or decreasing (blue) IgG reactivity in the four astronauts, detected using Peptide Set Enrichment Analysis^19^ (Methods). Shown are the four virus species for which at least one significant increase was detected with the six sampling time points indicated by vertical tick marks at the top of the plot. EBV, Epstein–Barr virus; EV-A, *Enterovirus A*; EV-B, *Enterovirus B*; EV-C, *Enterovirus C*; EV-D, *Enterovirus D*; HAdV-B, *Human mastadenovirus B*; HAdV-C, *Human mastadenovirus C*; HAdV-D, *Human mastadenovirus D*; HHV-1, *Human alphaherpesvirus 1*; HHV-4, *Human gammaherpesvirus 4*; HHV-5, *Human betaherpesvirus 5*; HHV-6, *Human betaherpesvirus 6*; HHV-7, *Human betaherpesvirus 7*; HOPV, *Human orthopneumovirus*; HRV3, *Human respirovirus 3*; HRV-A, *Rhinovirus A*; HRV-B, *Rhinovirus B*; HRV-C, *Rhinovirus C*; NV, Norwalk virus; RV-A, *Rotavirus*; SARS-CoV-2, Severe acute respiratory syndrome coronavirus 2.

The primary objective of the virome-wide antibody project was to assess immunological responses, including those that could be associated with viral reactivation, during short-duration spaceflight, as a multitude of evidence has demonstrated perturbation of the immune system (for example, decreased cellular immunity, dysregulation of T cell function and cytokine production) and reactivation of latent viruses during both short-duration (for example, space shuttle) and long-duration ISS missions^11–14^. Therefore, we generated virome reactivity profiles of the Inspiration4 crew across mission phases, leveraging a highly multiplexed assay (‘PepSeq’) that measures immunoglobulin G (IgG) reactivity to peptides representing the human virome^15–17^. PepSeq analysis using a previously described^15^ 15,000-peptide assay covering 80 human-infecting viral species showed that the reactivity profiles of dried blood spot samples collected in-flight clustered with those of terrestrial samples from the corresponding astronauts (Fig. 2a), indicating that the collection and storage of samples during flight did not adversely affect their quality. The time-invariant reactivity profiles of the four astronauts included reactivity to 45 species (Supplementary Table 3) and were dominated by reactivity to peptides from respiratory viruses and Epstein–Barr virus^13,18^, as expected (Fig. 2a).

To detect species-specific antibody changes during the sampling period, we conducted a peptide set enrichment analysis^19^ to compare all pairs of consecutive time points for each virus species (Fig. 2b). We detected a total of *n* = 8 such events (defined as significant reactivity increases); the largest number corresponded to Epstein–Barr virus (*n* = 4), a virus previously reported to be reactive in astronauts during short-duration spaceflight^13,18^. The remainder were species associated with common upper respiratory infections: *Rhinoviruses A* and *C* (*n* = 2 and *n* = 1, respectively) and *Adenovirus C* (*n* = 1). The Epstein–Barr virus events included changing reactivity that overlapped with flight for astronauts C001 and C002 during flight (Fig. 2b), although these specific viruses were not found in the sequence data (Fig. 1). These combined analyses indicate that changing immunity to viruses can be detected during spaceflight, even under conditions where no viral symptoms or replication are detected, which has been previously documented in astronauts during longer missions^14^.

## Point-of-care sampling and analysis

**Extended Data Fig. 3:**
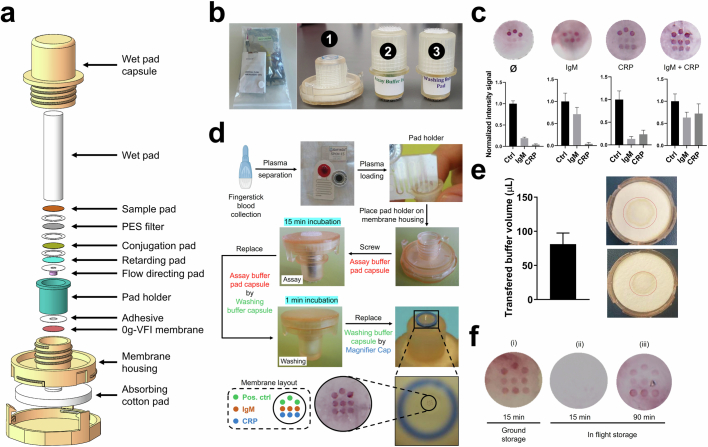
Paper-based multiplexed microgravity-adapted vertical flow assay (0g-VFI). **a**, Annotated exploded view of the 0g-VFI. PES, polyethersulfone. **b**, Pictures of the packaged 0g-VFI kit as stored in the Dragon (left) and the three different components of the 0g-VFI: (1) membrane housing with the magnifier cap, (2) assay buffer pad capsule and (3) washing buffer pad capsule. **c**, Schematic overview of the complete assay operation as performed during the mission, from the blood collection by fingerstick to the visual detection of the immunoglobulin M (IgM) and C-reactive protein (CRP) values, passing by the plasma separation using the Gattaco® cartridge and the different incubation and washing steps. **d**, Representative images of 0g-VFI membranes showing the multiplexing performance (top) and graphical quantification (bottom) of the values obtained when membranes were either incubated with no samples (ø), IgM only, CRP only or in multiplex with IgM + CRP (Data presented are mean ± SEM from three independent 0g-VFI (n = 3). **e**, Graph representing the average volume of assay buffer transferred to the absorbing pad when the 0g-VFI was used in microgravity during the I4 mission (Data presented are mean ± SEM; n = 3). Pictures show absorbing pads from two different 0g-VFI kits used in-flight with diameter of wet area (red circles), which informs the volume of assay buffer transferred from the wet pad during operation. **f**, Visual comparison of 0g-VFI membranes from kits stored in the lab and performed following standard protocol with 15 min incubation (i) with kits stored in the Dragon capsule and run in-flight for 15 min (ii) or post-flight for 90 min (iii).

**Extended Data Fig. 4:**
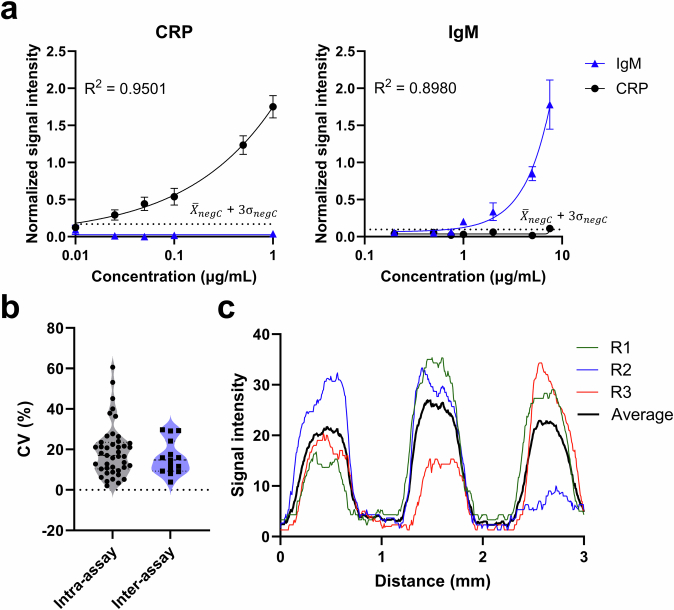
0g-Vertical Flow Immunoassay (VFI) analytical performance and quality control metrics. **a**, 0g-VFI intensity for C-reactive protein (CRP) and immunoglobulin M (IgM) spots are a function of CRP and IgM concentration on multiplex membranes. CRP and IgM samples were assayed from three independent 0g-VFI (n = 3) and each CRP and IgM data point presented is the mean ± SEM of the triplicate. For each CRP and IgM plot, a four-parameter logistic model was used to generate the fitted curve and the limit of detection (LOD), represented as a dotted line, was determined using the following formula: ${\rm{L}}{\rm{O}}{\rm{D}}={\bar{x}}_{{\rm{negC}}}+3{{\sigma }}_{{\rm{negC}}}$, where LOD is equal to the mean of the negative control (negC) + 3 standard deviations (σ) of the negative control. **b**, Intra- and inter-assay coefficients of variation (CV) across CRP and IgM assays are presented; CVs were calculated by CV (%) = (Standard Deviation/Mean) x 100. For the inter-assay CV, each point represents the CV of the control spots obtained from three independent identical replicates of 7 CRP and IgM 0g-VFI membranes (n = 14). For the intra-assay CV, each point represents the CV of the three control spots obtained within the same membranes from 43 independent CRP and IgM 0g-VFI. **c**, Representative intensity distribution across 0g-VFI membranes. The plot shows three IgM spots from three independent 0g-VFI membranes (R1, R2, and R3) exposed to an IgM concentration of 1 μg/mL; the average of the three IgM spots is presented in black.

**Extended Data Fig. 5:**
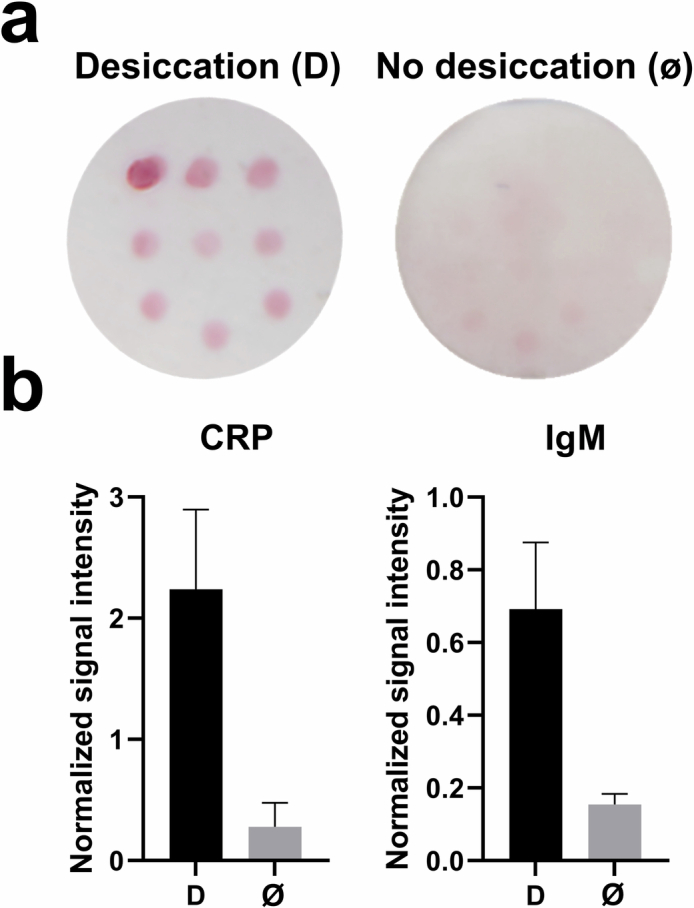
Accelerated stability studies of 0g-VFI devices. 0g-VFI devices were packed: 1) in the presence of a hygroscopic substance (Desiccation, D) or 2) no hygroscopic substance (No desiccation, ø) and stored in an environmental chamber at 42 °C and 75% humidity. **a**, Representative 0g-VFI membranes packed with (D) and without (ø) desiccation. **b**, C-reactive protein (CRP) and immunoglobulin M (IgM) quantification after a 3-day incubation. Data presented are mean ± SEM from three independent 0g-VFI (n = 3).

In addition to the viral and immune profiles, the Inspiration4 crew also tested a paper-based multiplexed microgravity-adapted vertical flow immunoassay (0*g*-VFI) (Methods and Extended Data Fig. 3a–c) to detect plasma immunoglobulin M (IgM) and C-reactive protein (CRP) in-flight via gold nanoparticle-conjugated antibodies. Assessment of cross-reactivity and specificity showed no significant non-specific binding, with a minimal background sometimes visible for IgM (Extended Data Fig. 3d). The limit of detection for CRP and IgM was 0.01 and 0.7 µg ml^−^^1^, respectively (Extended Data Fig. 4a). The intra- and interassay coefficients of variation were less than 20% (Extended Data Fig. 4b), and the presence of some high-level outliers was the result of a low spatial distribution homogeneity that was compensated by measurement repeatability (Extended Data Fig. 4c). Post-flight examination of the 0*g*-VFI showed that absorbing pads displayed a marked area, suggesting that an average of 80 µl of fluid crossed the membrane, thus highlighting both that fluid displacement occurred in the microgravity environment and that it was correctly used by the Inspiration4 crew (Extended Data Fig. 3e). Finally, the analysis of 0*g*-VFI membranes showed that no IgM or CRP were detected for the devices run in space following standard protocol (15 min incubation) by the crew (Extended Data Fig. 3f). Unused kits stored in the *Dragon* capsule were run post-flight on Earth and did not display any changes either. Furthermore, control kits stored on Earth with hygroscopic compounds for the same period (that is, the duration of the flight experiment) and run for 15 min also showed strong intensity, suggesting the need to maintain low humidity levels during storage. Accelerated stability studies conducted in an environmental chamber at 42 °C and 75% humidity confirmed the effect of desiccation on VFI performance, with a more than 8-fold decrease in intensity after storage without a hygroscopic compound (Extended Data Fig. 5).

## Ultrasound imaging of the Inspiration4 crew

**Fig. 3:**
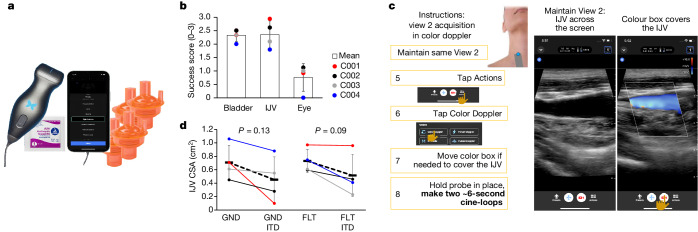
Imaging-based experiments. **a**, Payload: Butterfly iQ+ ultrasound system and ResQGARD ITD7 used for physiological intervention. **b**, Summary plot of image quality assessment. For each image acquired, imaging success scores were calculated based on anatomical accuracy and technical quality (Methods). The mean success score for each astronaut is plotted as an individual data point, and grey-dotted bars represent the grand mean ± s.d. of astronaut success scores for each anatomical target; *n* = 4 astronauts, *n* = 14 bladder images, *n* = 73 IJV images and *n* = 19 eye images. Eye imaging success scores were different than bladder and IJV scores per one-way ANOVA and post hoc Tukey’s honest significant difference. **c**, A sample page from the JIT instructions for flow spontaneity assessment. **d**, CSA of the right IJV pre-flight in the supine position (GND) and in-flight (FLT), with and without ITD breathing. Pre-flight data were derived from a single imaging instance per astronaut, while in-flight data were averaged across in-flight instances within each astronaut (range = 1–3 imaging instances); mean IJV CSA for each astronaut is presented for each condition, along with the grand mean ± s.d. The difference between grand means for each IJV CSA assessment pre-flight (GND) and in-flight (FLT) is visualized with a black dashed linear trend line; *n* = 4 astronauts, *n* = 4 GND images, *n* = 4 GND ITD images, *n* = 8 FLT images and *n* = 7 FLT ITD images. No differences between pre-flight (GND) and in-flight (FLT) were found evaluated using paired, two-tailed Student’s *t*-tests with a significance threshold of *α* < 0.05.

Traditional imaging equipment and techniques, while established aboard the ISS, may not be afforded in resource-constrained missions due to their size and resource dependence. The Inspiration4 mission featured the first in-flight research use of Butterfly iQ+, a handheld single-probe ultrasound device for urinary bladder, internal jugular vein (IJV) and eye imaging in full crew autonomy (Fig. 3). Aims for all targets included both the assessment of physiological changes in response to spaceflight and evaluation of autonomous procedure efficacy. Eighty-nine imaging instances (multiframe cine of varied length) were collected pre-flight, and 108 imaging instances were collected in-flight, yielding an average of 27 instances per astronaut (range = 18–32 instances). Bladder imaging success scores were above the usability threshold (2.33 ± 0.24 (s.d.); range = 2.0–2.5; Methods), demonstrating the effectiveness of the imaging system and the instructions for urinary bladder volume assessments (Fig. 3b). However, these image sets did not reveal significant physiological trends, possibly due to variability between the small number of astronauts and crew-reported operational constraints affecting the timing of data acquisition relative to voiding. The bilateral IJV images were the highest in quality (2.36 ± 0.51 (s.d.); range = 1.8–2.9) and thus amenable to quantification and analysis. The in-flight image sets from ocular imaging (the most intricate procedure) consistently scored below the usability threshold in all four astronauts (0.76 ± 0.51 (s.d.; range = 0.0–1.1); the mean ocular success score was lower than both the bladder and IJV success scores, which were of similar quality. Therefore, ocular imaging data were deemed inadequate for quantification of microgravity-induced structural changes.

The IJV imaging protocol was similar to the protocol described in ref. ^20^, which was designed to measure bilateral IJV cross-sectional areas (CSAs) and flow velocities, and can be used to identify anomalies, such as thrombi, loss of flow spontaneity, flow reversal and spontaneous echo contrast development^21,22^ (Fig. 3c). In contrast to long-duration ISS cohorts^20,22^ and regardless of flight day (FD1–3), both IJVs were free from thrombi and flow anomalies in all Inspiration4 astronauts. Spontaneous antegrade flow was demonstrated in all examined IJVs by colour and (or) spectral Doppler and no spontaneous echo contrast was detected. While previous studies of astronauts in both short-duration (less than 7 days) and long-duration spaceflight have demonstrated a stable increase in IJV CSA^20,23,24^, the absence of flow anomalies and spontaneous echo contrast in all four astronauts suggests a potential difference from respective long-duration spaceflight data and the possibility of lower risk of IJV thrombosis in the early period of microgravity exposure. The effect of inspiratory resistance breathing using an impedance threshold device (ITD) on IJV filling (reflected as CSA) appeared more pronounced on the right side, but varied among astronauts and did not reach statistical significance (Fig. 3d, *t*-test, *P* values > 0.05). Notably, the measurements were concordant among astronauts pre-flight and in-flight (Fig. 3d), indicating reliable data collection and encouraging further inquiries into the potential of ITD-like interventions as a countermeasure against thrombosis in cases of clinical concern with commensurate flow anomalies.

## Otolith asymmetry and motion sickness

**Fig. 4:**
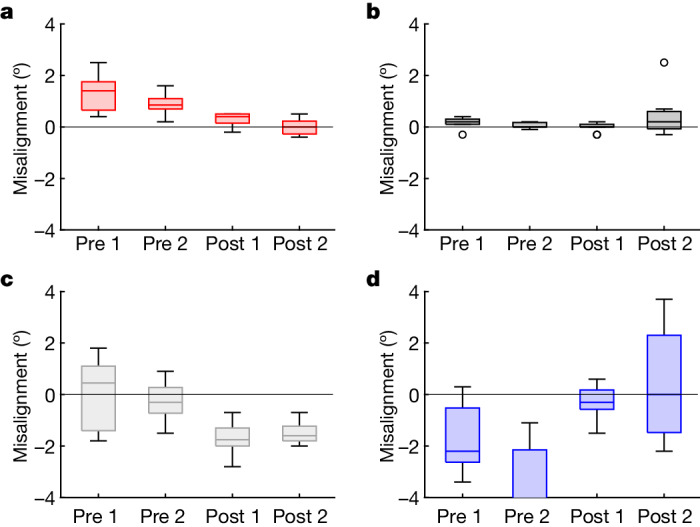
Ocular misalignment before and after short-duration spaceflight. **a**–**d**, The response of the neurovestibular system to short-duration spaceflight was indexed by ocular misalignment, as a proximal measure of otolith asymmetry. The degree of vertical ocular misalignment (VAN) is shown for each of the *n* = 4 astronauts as follows: C001 (**a**), C002* (**b**), C003 (**c**), C004* (**d**). An asterisk denotes astronauts who reported SMS in-flight; in these astronauts (C002 and C004), VAN scores were not significantly different post-flight relative to pre-flight. Each box represents one test session (*n* = 2 pre-flight and *n* = 2 post-flight, for each astronaut), in which 11 VAN trials were performed. The horizontal bar in each box represents the median of that dataset, the box encompasses the central 50% of the dataset and the whiskers indicate the minimum and maximum values that are not outliers (outliers, which are more than three scaled median absolute deviations from the median, are indicated by circles). Two-sample two-tailed *t*-tests were performed for each astronaut individually to determine consistency of pre-flight and post-flight measures, and significant differences (indicating spaceflight adaptation) between pre-flight and post-flight measures.

**Extended Data Fig. 6:**
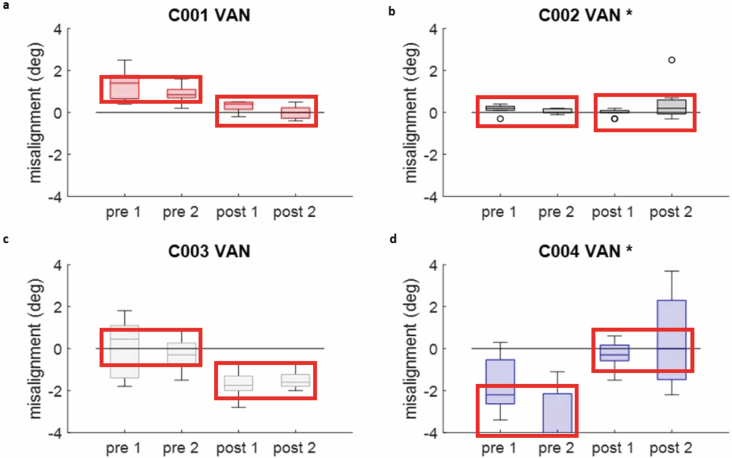
Ocular misalignment before and after short-duration spaceflight. Data as presented in Fig. 5. Red boxes indicate the grouping, for each astronaut, of pre-flight and the post-flight tests; an asterisk (*) denotes astronauts who experienced space motion sickness (SMS) in-flight. This shows consistency of pre-flight and post-flight tests in the non-SMS astronauts (C001 and C003). Vertical offsets of the boxes in each graph show change from pre-flight to post-flight, indicative of adaptive change during flight (larger adaptive change associated with lack of SMS). Boxes are drawn manually, to enclose the median in each case.

The neurovestibular system is adversely affected by the microgravity environment of space through altered neural processing and transduction of sensory measurements^25,26^. The otolith organs are an integral component of the neurovestibular system that transduce sensory effects, particularly linear acceleration and gravity. Any normal asymmetry between otoliths on the two sides of the head is compensated for by central neural processes on Earth^27^; however, this compensation is inappropriate in microgravity, which can lead to vertical and torsional ocular misalignment. This asymmetry (as manifest in ocular misalignment) has also been associated with increased susceptibility to space motion sickness (SMS)^25^. Ocular alignment was assessed in the Inspiration4 crew, as a proximal measure of otolith asymmetry, during the pre-flight and post-flight phases using a computerized test that measured vertical (vertical alignment nulling (VAN)) and torsional (torsional alignment nulling (TAN)) ocular misalignment. VAN and TAN data collected on the Inspiration4 crew showed no consistent and systematic effects of spaceflight from pre-flight to post-flight. VAN, however, exhibited different patterns between astronauts who experienced SMS in-flight compared to those who did not (Fig. 4). Two of the four Inspiration4 crew (50%) experienced SMS, which is consistent with earlier reports that 50–67% of astronauts experience SMS during short-duration spaceflight^28,29^. Astronauts who did not experience SMS (Fig. 4a,c) exhibited consistent vertical misalignment both pre-flight and post-flight, and that misalignment was different after the flight than before the flight. This is presumably because post-flight testing reflects the in-flight adaptive state, and that 0*g*-adapted state should, in general, be different from the 1*g* state (*P* values < 0.001; Supplementary Table 4 and Extended Data Fig. 6). A statistical evaluation of the proposed interpretation of SMS susceptibility was developed to validate these observations (Supplementary Information).

## Cardiovascular responses to spaceflight

**Fig. 5:**
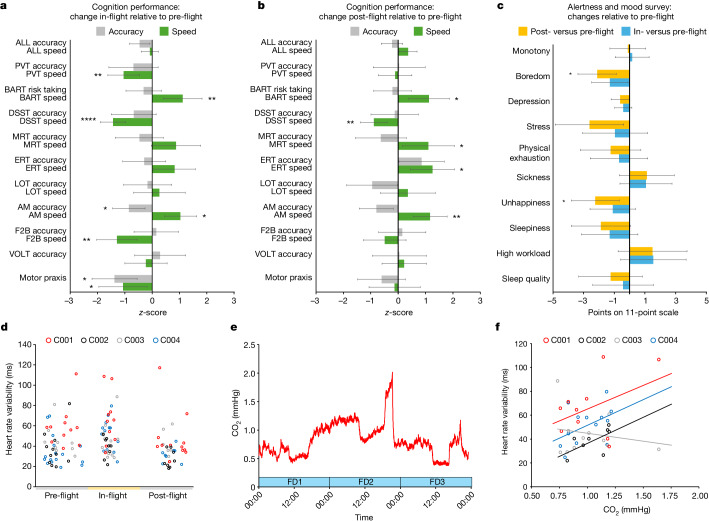
Behavioural and physiological responses to short-duration spaceflight. **a**,**b**, The standardized difference in accuracy (grey) and speed (green) of astronaut (*n* = 4) cognitive performance on the ten assays of the cognition test battery (*n* = 26 administrations) and unadjusted 95% confidence intervals. Response speed and accuracy metrics were standardized (*z*-scored) before analysis to allow for comparison among cognitive domains. **a**, Difference in cognition accuracy and speed in-flight relative to pre-flight. **b**, Difference in cognition accuracy and speed post-flight relative to pre-flight. **c**, Change in astronaut ratings of their behavioural state in-flight (blue) and post-flight (orange) relative to pre-flight and unadjusted 95% confidence intervals. Astronauts reported on their behavioural state using 11-point Likert scales using the alertness and mood survey^50^. For **a**, **b** and **c**, differences between mission phases were tested using mixed-effect models contrasting in-flight and post-flight relative to pre-flight; *P* values were corrected for multiple comparisons using the false discovery rate method^51^, and adjusted significant associations are denoted as: *P < 0.05; **P < 0.01; ****P < 0.0001. **d**, Average HRV, a measure of parasympathetic regulation of cardiac function, across 1 h measurement periods (*n* = 127). HRV estimates were derived from heart period data collected via the Apple Watch using the s.d. of RR intervals. Significant changes in HRV (*F* = 5.64, *P* = 0.0046) and heart rate (*F* = 37.10, *P* < 0.0001) were observed in the Inspiration4 crew across mission phases. **e**, One-minute averages of spacecraft CO_2_ levels. NASA’s current 1 h standard restricts CO_2_ levels to less than 3 mmHg. **f**, Relationship between 1 h average CO_2_ levels in the spacecraft and HRV for each astronaut in-flight (*n* = 44). AM, abstract matching; BART, balloon analogue risk test; DSST, digit–symbol substitution task; ERT, emotion recognition test; F2B, fractal 2-back; LOT, line orientation test; MP, motor praxis task; MRT, matrix reasoning test; PVT, psychomotor vigilance test; VOLT, visual object learning test.

**Extended Data Fig. 7:**
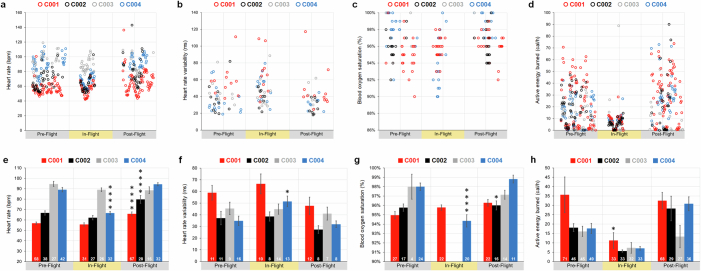
Cardiovascular responses to short-duration spaceflight. **a**, One-hour averages of astronaut heart rate (HR) measured across all mission phases (N = 424). **b**, One-hour averages of astronaut heart rate variability (HRV), a measure of parasympathetic regulation of cardiac function, derived from heart period data using the standard deviation of R-R intervals across mission phases (N = 127). **c**, Scatter plot of astronaut blood oxygen saturation across mission phases (N = 177); blood oxygenation measurements failed in two astronauts in-flight. **d**, Scatter plot of one-hour averages of astronaut energy consumption across mission phases (N = 502). **e**, Average hourly HR for each mission phase. **f**, Average hourly HRV for each mission phase. **g**, Average hourly blood oxygen saturation for each mission phase. **h**, Average hourly energy consumption for each mission phase. For panels **e**-**h**, mean ± SEM with the *n* for each astronaut during that mission phase. Differences in cardiometabolic measures in-flight and post-flight relative to pre-flight were evaluated using mixed-effect models that nested repeated measures within astronauts contrasting the in-flight and post-flight periods with pre-flight; P*-*values were corrected for multiple comparisons using the false discovery rate method^51^ and adjusted significant associations are denoted as: *P < 0.05; ****P < 0.0001.

While some wearable devices have previously flown in other spaceflight missions, crew of the Inspiration4 mission used Apple Watches, which feature a rich set of biometrics that can be collected on astronauts and have the potential to be useful in future missions. Astronaut cardiovascular physiology was measured with the Series 6 Apple Watch, which astronauts donned on FD2 and wore for an average of 1.3 ± 0.1 days in-flight (Extended Data Fig. 1). Significant changes across mission phases were observed for crew heart rate (Extended Data Fig. 7a,e) and heart rate variability (HRV; Fig. 5d and Extended Data Fig. 7b,f), and differential heart rate changes between astronauts were also observed (*F* = 9.46, *P* < 0.0001). Studies of heart rate in spaceflight have yielded mixed findings^30^, and relative to pre-flight, only C004 exhibited decreased heart rate (Supplementary Table 5), as well as increased HRV (Fig. 5d). These cardiovascular changes in C004 were accompanied by lower blood oxygen saturation in-flight (Extended Data Fig. 7c,g), yet post-flight measures of cardiovascular function did not differ from baseline (pre-flight) upon return to Earth. Consistent with earlier studies^31^, increased heart rate post-flight, relative to pre-flight, was observed for two astronauts, C001 and C002; C001 also exhibited elevated blood oxygen saturation levels post-flight. Furthermore, the crew exhibited substantially lower overall activity in-flight relative to pre-flight (*F* = 6.65, *P* < 0.0001), which was accompanied by reduced active energy expenditure (Extended Data Fig. 7d,h; *F* = 9.29, *P* = 0.0001); this reduced energy expenditure was driven by C001.

**Extended Data Fig. 8:**
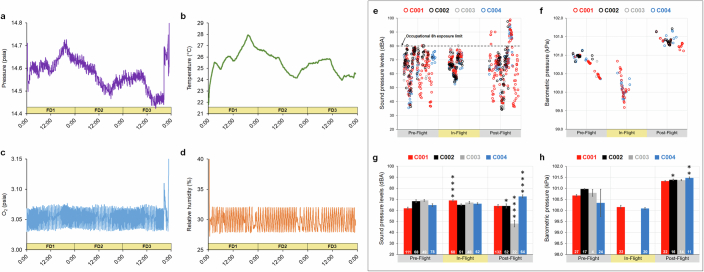
Profiles of the spacecraft environment during short-duration spaceflight. **a**, One-minute averages of cabin pressure of the spacecraft measured throughout the Inspiration4 mission (FD: flight day). **b**, One-minute averages of temperature in the spacecraft. **c**, One-minute averages of partial pressure of Oxygen (O_2_) in the spacecraft. **d**, One-minute averages of relative humidity in the spacecraft. **e**, Scatter plot of sound pressure levels for each astronaut, measured using the Apple Watch, across mission phases; sound pressure levels were averaged arithmetically, and each point represents a one-hour average (N = 797). **f**, Scatter plot of barometric pressure levels for each astronaut, measured using the Apple Watch, across mission phases; each point represents a one-hour average (N = 177). **g**, Average sound pressure level for each astronaut during each mission phase (mean ± SEM), which was sampled twice per hour by the Apple Watch (N = 797). Sound pressure levels were similar in-flight relative to pre-flight for three of the four astronauts; one astronaut was exposed to higher sound pressure levels (C001; β = −7.152; P < 0.0001) in-flight. **h**, Average barometric pressure level for each astronaut during each mission phase. For panels **g** and **h**, mean ± SEM with the *n* for each astronaut during that mission phase. Differences in-flight and post-flight relative to pre-flight were evaluated using mixed-effect models contrasting the in-flight and post-flight periods with pre-flight; post-hoc analyses testing for differences between astronauts were corrected for multiple comparisons using the false discovery rate method^51^. Significant associations are adjusted for multiple comparisons: *P < 0.05; **P < 0.01; ****P < 0.0001.

Some of the effects of spaceflight on astronaut physiology and behaviour can be attributed to the spacecraft environment^32^. The cabin environment was measured via sensors in the spacecraft, while sound pressure levels were measured using the Apple Watch. Environmental variables fluctuated over time in-flight (Extended Data Fig. 8 and Supplementary Table 5). The Inspiration4 crew were confined to the *Dragon* capsule, which has a volume of 9.3 m^3^, and were free to move about the cabin. The *Dragon* cabin environment is nominally controlled to 14.7 psi and 21% O_2_, in-flight O_2_ partial pressures averaged 3.1 psia (range = 3.0–3.3 psia; equivalent to 20.4–22.4%) and CO_2_ averaged 0.8 mmHg (range = 0.4–2.0 mmHg). Temperature and humidity are controlled to maintain a comfortable short-sleeve environment; the crew had the capability to adjust the temperature, which averaged 25.5 °C (range = 21.8–27.9 °C) in-flight. Relative humidity could range between 25% and 75% (typically around 50%) and averaged 29.9% (range = 27.3–52.3%). The *Dragon* environmental conditions were equivalent to those on the ISS, with O_2_ ranging from 2.8 psia to 3.0 psia, temperature ranging from 18 °C to 25 °C, relative humidity of 25–75% and a cabin pressure ranging from 3.8 psia to 14.9 psia. Aside from one period with high sound pressure levels in three astronauts post-flight, sound pressure levels were similar across mission phases; of note, the spacecraft produced around 50 dBA of background noise. Noise exposure remained below the occupational 8 h exposure limit of 80 dBA for all but one data point (Extended Data Fig. 8e,g). In-flight, three of the four astronauts exhibited a significant and positive association between CO_2_ levels and higher HRV (Fig. 5f); however, other spacecraft environmental factors, including cabin pressure and temperature, did not exhibit consistent relationships with HRV.

## The Inspiration4 crew’s neurobehavioural functions

**Extended Data Fig. 9:**
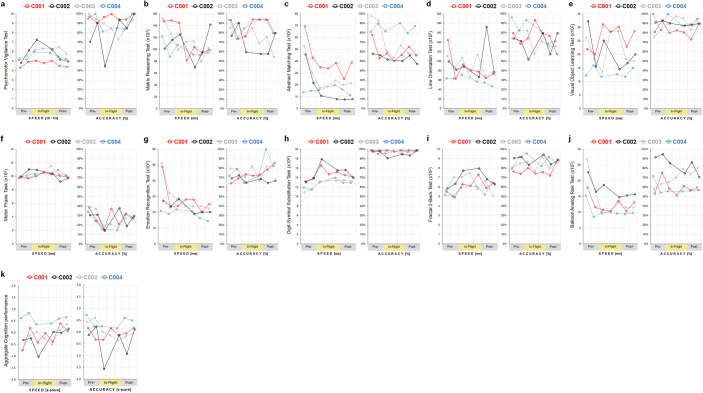
Profiles of astronaut cognitive performance on *Cognition* assays across mission phases. Performance on each of the 10 *Cognition* tests is indexed by response speed in the left panel and accuracy in the right panel for each astronaut across mission phases (N = 26 *Cognition* administrations). For response speed outcomes, higher values represent slower response speeds; response speed on the Psychomotor Vigilance Test is inverted to match the direction of other Cognition speed metrics. For accuracy outcomes, higher values represent more accurate performance. **a**, Psychomotor Vigilance Test (PVT). **b**, Matrix Reasoning Test (MRT). **c**, Abstract Matching (AM). **d**, Line Orientation Test (LOT). **e**, Visual Object Learning Test (VOLT). **f**, Motor Praxis Task (MP). **g**, Emotion Recognition Test (ERT). **h**, Digit-Symbol Substitution Task (DSST). **i**, Fractal 2-Back (F2B). **j**, Balloon Analog Risk Test (BART). **k**, Aggregate Cognition speed and accuracy across tests (data for each test were standardized before averaging; BART risk taking does not contribute to the accuracy score across tests).

Astronaut neurocognitive functioning and behavioural health are integral to the success of spaceflight missions. Astronauts performed the ten cognitive tests of NASA’s Cognition battery^33^ multiple times during each mission phase (Fig. 5a–c and Extended Data Fig. 1). Across cognitive domains and as consistent with earlier work^34^, astronaut cognitive performance was largely unaffected by short-duration spaceflight (Supplementary Table 6). Astronauts were, however, significantly slower on four cognition tests, and on three of them (psychomotor vigilance test, digit–symbol substitution task and motor praxis task assessing sustained attention, visual search and working memory and sensorimotor speed, respectively), astronauts were also less accurate (albeit statistically significantly so for the motor praxis task only), suggesting lower cognitive efficiency (Fig. 5a and Supplementary Note 3). These cognitive deficits were partially driven by one astronaut (C002), who exhibited a substantial performance deficit early in-flight (Extended Data Fig. 9). Except for the digit–symbol substitution task, cognitive performance post-flight did not differ from performance pre-flight (Fig. 5b and Supplementary Table 6).

**Extended Data Fig. 10:**
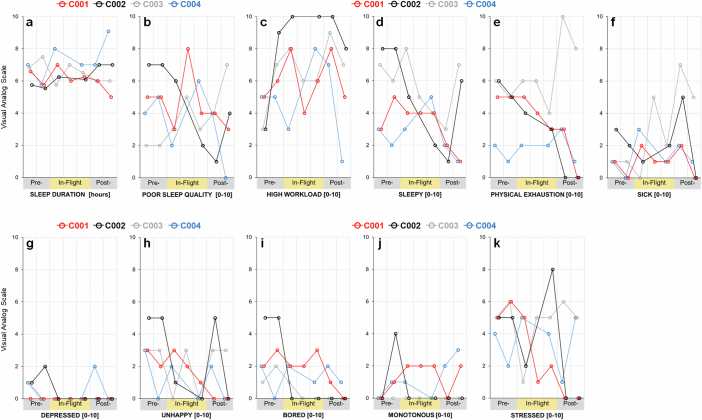
Astronaut report of behavioral states across mission phases. Astronauts (N = 4) reported on their sleep duration and behavioral state using the Alertness and Mood Survey (AMS)^50^. For all 10 AMS items (panels **b**-**k**), astronauts rated their behavioral state using 11-point Likert-type scales for each item during all three phases of the mission (N = 26 for each item). AMS items are plotted on the full range of each scale and higher numbers represent higher ratings in the direction of the item at the bottom of each panel (e.g., for Poor Sleep Quality, a higher number represents worse sleep quality). For all AMS items except sleep quality and workload, astronauts were prompted to rate “how are you feeling right now?” **a**, Sleep duration (derived from self-reported sleep and wake-up times). **b**, Sleep quality (question: “How was the quality of your sleep?”; anchors: good—poor). **c**, High workload (question: “What was today’s workload?”; anchors: very low—very high). **d**, Sleepiness (anchors: not sleepy at all—very sleepy). **e**, Physical exhaustion (anchors: energetic—physically exhausted). **f**, Sickness (anchors: healthy—sick). **g**, Depressed (anchors: not depressed at all—very depressed). **h**, Unhappiness (anchors: happy—unhappy). **i**, Bored (anchors: good—poor). **j**, Monotonous (anchors: good—poor). **k**, Stressed (anchors: not stressed at all—very stressed).

Astronauts also completed an alertness and mood survey (AMS) before performing each Cognition battery. Nightly sleep duration of 6.7 ± 0.7 h in-flight was reported, which is modestly longer than previous studies^35,36^. Astronauts reported a moderate level of stress and high workload, similar to astronauts completing 6-month ISS missions^36^, but reported no other alertness or mood symptoms (Extended Data Fig. 10). In-flight mood and alertness did not differ from pre-flight, but astronauts reported being significantly happier and less bored post-flight (Fig. 5c and Supplementary Table 7). Although astronaut reports of adverse behavioural states were overall similar across mission phases, there was substantial variability both within and between astronauts in their behavioural responses to spaceflight (Extended Data Fig. 10). Furthermore, one astronaut (C003) exhibited a considerable increase in physical exhaustion upon return to Earth; overall, these behavioural responses to short-duration spaceflight are consistent with a previous report of astronauts in long-duration (6-month missions) spaceflight^36^.

## Discussion

To understand the effects of short-duration spaceflight on the human body for the all-civilian spaceflight crew, this study performed a suite of experiments and collected a wide range of biospecimens before, during and after a 3-day orbital mission. This multidimensional battery included blood, stool, urine, biopsy and saliva samples, ultrasound measurements of the eye, jugular vein and bladder, cognitive and sensorimotor tests, surveys and physiological data collected with an Apple smartwatch. These data showed some of the same signatures of long-duration spaceflight, such as inflammatory response, DNA damage response gene expression (and proteins), telomere elongation and immune signalling changes (Figs. 1 and  2), and demonstrated that such phenotypes can also be observed in the earliest phases of spaceflight and across a shorter mission time frame. Moreover, this mission also enabled new biomedical metrics for spaceflight, such as RNA methylation, single-nucleus chromatin, single-cell expression metrics and spatial transcriptomics. Overall, this work demonstrated that a diverse civilian crew can conduct scientific experiments, process samples and significantly contribute to spaceflight research with minimal risk. Although two astronauts presented with SMS, most metrics (for example, IJV size, heart rate, complete blood count metrics, gene expression and cytokines) were either stable or quickly reverted to pre-flight levels (baseline) after landing on Earth.

The development of diagnostic, point-of-care devices that can detect and quantify multiple biomarkers is critical to monitor astronauts’ healthcare for future spaceflight missions and to help guide medical interventions. This mission showed that a VFI can be used to detect antigens and, more generally, protein markers^37–39^. Due to flight certification constraints required for all technologies used in spaceflight, the desiccant usually placed in the packaging kit to avoid high humidity exposure during long-term storage had to be removed, and has been identified as the main cause for the alteration of the assay efficiency in-flight. For future missions, reagent and system stability studies will have to determine optimal storage conditions without the use of hygroscopic compounds. Despite this obstacle, the 0*g*-VFI demonstrated its robustness of operation in space, and the simplicity of its user interface allowed for its use by untrained non-scientists, supporting the feasibility of such point-of-care diagnostic systems for several applications on additional specimen types, including for deep-space missions.

The Inspiration4 ultrasound imaging experiment pursued three research objectives, which were to examine (1) urinary bladder function in microgravity, (2) IJV flow with and without intervention by inspiratory resistance and (3) microgravity-associated changes in ocular morphology. The choice of targets was motivated by high-priority concerns and risks reported over the years of ISS missions. For example, flow anomalies in the IJV (for example, severe stasis, flow reversal) frequently develop in ISS crew members in the later stages of long-duration spaceflight^20–22^; however, such alterations in cardiovascular function have been less studied in the earlier days of spaceflight when precursors to cardiovascular risks may begin to emerge^23^. In the Inspiration4 mission, IJV flow anomalies were not observed in any of the four astronauts, suggesting that flow anomalies develop later in-flight. The lack of IJV flow anomalies also diverge from Earth-based simulations of microgravity (head-down tilt), which demonstrate IJV and other vascular alterations after five days of exposure^23^. If corroborated by future studies in short-duration spaceflight, this finding may further our understanding of the mechanisms and progression pattern of flow degradation in the left IJV during sustained microgravity. Inspiratory resistance reduced right IJV CSA by an average of 36% pre-flight (supine) and 29% in-flight but did not reach statistical significance in this small cohort of astronauts. This trend suggests facilitated IJV drainage due to resistance breathing, and the experiment demonstrated a simplified in-flight screening method, with resistance breathing as an option to correct anomalous IJV flow. Overall, these findings bode well for the cardiovascular safety profile of short-duration missions, although future studies that address other anatomical targets are needed, as the effects of spaceflight are not uniform throughout the cardiovascular system^23,24^. A better understanding of the cardiovascular risks could alleviate some of the spaceflight health concerns for private citizens with diverse health backgrounds and medical histories, although notably, no health issues were observed in the Inspiration4 crew.

The Inspiration4 mission undertook ultrasound imaging-based human research in full crew autonomy, with minimal pre-flight familiarization and reliance on experiment-specific just-in-time (JIT) instruction. Probing the potential limitations of miniaturized ultrasound technology combined with the rapid deployment and complete crew autonomy was an inseparable part of the experiment. This autonomy is in stark contrast to the ISS experience, where all imaging sessions are conducted with a traditional device and in real-time interaction with experts in the mission control centre. The anatomical and technical quality of the images, expressed as overall success scores, appeared inversely related to the complexity of the autonomous procedures. Bladder imaging (the simplest procedure) and IJV imaging (intermediate difficulty and complexity) each had similar scores, well above the usability threshold. Conversely, the ocular imaging procedure (most intricate), which required precise gaze control and accurate probe manipulation by real-time visual feedback, did not produce image sets of sufficient quality to derive the intended measures, such as globe axial length or optic nerve sheath diameter. In this study, all but the most intricate procedures could be successfully performed by minimally trained users with the aid of appropriate JIT materials. Based on these findings, as well as those acquired during similar applications on the ISS, ocular imaging with JIT instruction in future missions should involve a dedicated ultrasound operator; however, other exams can be reliably performed in self-scanning, autonomous mode, which represents a significant medical and research capability in situations with degraded communications (for example, high latency, loss of communication). Payloads consisting of a miniaturized imaging system with an intuitive interface can enable future experiments featuring short lead and training times, rapid deployment, flexible schedules and data collection autonomy, which will enable scientific research opportunities even on the most constrained of spaceflight missions.

Studying ocular misalignment and otolith symmetry in short-duration spaceflight is not without caveats. First, the Inspiration4 results (Fig. 4) are based on comparing pre-flight and post-flight tests and relating that comparison to in-flight SMS. The ultimate goal is to use pre-flight tests alone to predict in-flight susceptibility, which is planned for future flights. The incidence of SMS was 50% in the Inspiration4 mission, which is consistent with the reported incidence in short-duration spaceflight^29^. Second, there is no relation between motion sickness susceptibility in parabolic flight and susceptibility in orbital flight, yet early work on torsional alignment was performed in parabolic flight to predict susceptibility in orbital flight^40^. Third, our results with VAN are promising for SMS prediction, while results with TAN are not (of note, the original work^41^ relating SMS to ocular skew was done with torsion). Finally, SMS has multiple contributing factors and multiple manifestations that vary between individuals, and thus it may be unrealistic to think that a single measure will continue to have high predictive power in a larger population.

Consistent with studies of astronauts and cosmonauts completing both short- and long-duration spaceflight missions^34–36^, collecting objective measures of astronaut cardiovascular functions and neurocognitive functioning, as well as subjective measures of sleep and astronaut behavioural states, is feasible in commercial astronaut crews throughout short-duration orbital missions. The Inspiration4 crew provided valid and usable data on cardiovascular physiology, cognitive performance and behavioural states across mission phases that can translate to the general public^3,42^, which is important as trained astronauts from international space agencies are not necessarily representative of the average individual. The effects of short-duration spaceflight on Inspiration4 crew cardiovascular physiology and cognitive performance were modest, although there was substantial interindividual variability in the response to spaceflight, as previously observed^34,43,44^.

Studies of cognitive performance have generally found that astronauts maintain relatively high levels of performance in spaceflight, although this may be a function of the duration of spaceflight missions, where performance decrements manifest with longer mission durations^1,34^. In this study, accuracy on eight of the ten (80%) cognition tests was unaffected by short-duration spaceflight (Fig. 5). There was, however, more variability in astronaut response speed on neurocognitive tasks, where slower response speeds were observed on four cognition tests, a finding consistent with earlier reports of astronaut response slowing during short-duration spaceflight (that is, less than or equal to 10 days in spaceflight shuttle missions)^45,46^.

Although previous studies have not found large cognitive performance deficits in spaceflight, this may be due to the timing of neurocognitive testing, which often does not occur on the first day in-flight, but up to four days in-flight, at which point cognitive deficits may have dissipated. Furthermore, the observed changes in response speed may, to some extent, be associated with neurovestibular and sensorimotor alterations induced by spaceflight^26,47^. While astronaut sleep durations less than 6 h on the ISS have been associated with psychomotor slowing, Inspiration4 astronauts averaged 6.7 h of sleep per night in-flight and thus the contribution of sleep loss to slower response speeds would be modest if present^36^. Astronauts did not report overtly negative behavioural states in-flight (for example, depression, stress), but they reported more happiness and less boredom post-flight relative to pre-flight (Fig. 5).

Significantly, all data from this study are stored in multiple controlled-access repositories for easy access, including TrialX, NASA’s Open Science Data Repository and GeneLab, the SOMA data portal and the Commercial Spaceflight Data Repository, which facilitates the collection of mission-specific research, medical data and biospecimens to be stored in a permanent electronic data and tissue repository for future scientific research. These data also include qualitative survey data received via mobile apps, DICOM-standard images from portable ultrasound devices, data from wearables (for example, heart rate, electrocardiogram), biospecimen information, cell processing details^48^ and the environmental/mission data from the *Crew Dragon* capsule (for example, cabin pressure, temperature and humidity, spacecraft telemetry), which can help guide exploration-class future missions^49^. Finally, the repository contains a visualization dashboard for researchers to view summary analytics and access individual observations and data files, which has been added to TRISH’s enhancing exploration platforms and analogue definition programme.

Although the Inspiration4 mission was the first all-civilian crew to complete a short-duration, orbital spaceflight mission while collecting repeated multidimensional measurements of biological and behavioural function relevant to the challenges of spaceflight, the study is not without limitations. Although the total sample of *n* = 4 astronauts is small, it is not inconsistent with previous studies of astronaut cognitive performance in spaceflight. While data collection spaceflight is challenging and limited by numerous factors (for example, cost, limited time and access to astronauts), longitudinal sampling of measures within and across mission phases can promote statistical power (Supplementary Note 1). This study did not have age- and sex-matched controls on Earth; however, repeated measures within the Inspiration4 crew across mission phases allow for astronauts to serve as their own controls (within-subjects design) by using their pre-flight (that is, baseline) levels as a reference. Future studies, or additional studies with equivalent design, of civilian astronauts in short-duration spaceflight with larger sample sizes are needed to confirm the findings or to assign any possible causal links.

Finally, it is worth noting that the Inspiration4 mission was not designed to address how the biological and behavioural responses of the Inspiration4 crew relate to those of professional astronauts or cosmonauts, or those with career-long exposure to spaceflight. Furthermore, the research on the Inspiration4 mission was not designed to determine the safety of spaceflight for all civilians, or to recommend spaceflight for future civilian passengers; the Inspiration4 research projects were also not tasked with making judgements for future crew selection or fitness for spaceflight. The selection of astronauts for spaceflight is exclusive to international space agencies (governmental or commercial). Nonetheless, the Inspiration4 data, along with data from other civilian spaceflight missions, may contribute to the development of reference ranges and pre-flight preparatory tasks (for example, behavioural testing in confined environments) that can help guide future crew selection and mission planning.

## Conclusions

While broad in its research scope, this study represents only the beginning. Anatomical and physiological variability, small sample size, the operational complexities of a highly constrained mission and limited skill management capabilities combined to preclude confident conclusions on many physiological variables. However, as intended by the TRISH, enhancing exploration platforms and analog definition, and SOMA programmes, these Inspiration4 data will serve as a rich foundation for scaling and enhancing the knowledge base on early phases of space physiology, and expanding our understanding of spaceflight-associated effects on human health. Future missions can also include telemedicine, more autonomous data collection, next-generation sequencing-based in-flight omics assays and related diagnostics tools. Excitingly, some of these same astronauts will be present on future missions and/or contribute to long-term studies of astronaut health, which will help delineate the short- and long-term impacts of spaceflight and continue to prepare future astronauts for their missions.

Finally, it is worth noting that collaboration with government, academia and the private sector at the same time led to lessons learned that should be useful to investigators embarking on similar endeavours in future civilian spaceflight missions. Specifically, co-ordination among diverse research teams can be enabled by agile funding entities (for example, TRISH, SpaceX, philanthropy) and a dedicated project manager. Also, a single institutional review board (IRB) protocol, such as that put in place by TRISH, can simplify subject consenting, while a standardized database provider (TrialX, funded by TRISH) was critical for data ingestion, cross-institute sharing and standardization for future cross-mission analyses and use of artificial intelligence and machine learning approaches. Finally, the consent and release forms of study participants were envisioned for long-term use, with datasets managed under the oversight of a data release board, and for these precious samples to be utilized and characterized for many years to come.

## Methods

### Subjects and consent

Four adult non-professional astronauts were selected to participate in the Inspiration4 mission. All subjects provided written informed consent to participate in the study, which included the collection and use of their samples and data in research protocols at Weill Cornell Medicine, Baylor College of Medicine/TRISH and collaborating institutions; the study was approved by multiple IRBs and included the following IRB protocols: Weill Cornell Medicine IRB no. 21-05023569, WCG-IRB 1309934, Multimodal Evaluation of Spaceflight Participants Health – SpaceX Inspiration-4 Mission; Baylor College of Medicine/Translational Research Institute for Space Health IRB no. 1316696, WCG-IRB 20214456, Commercial Spaceflight Data Repository. Subjects consented to the storage of their de-identified, coded research data in a secure, password-protected database at SpaceX, Weill Cornell Medicine, Baylor College of Medicine/TRISH, and the institutions of study co-investigators. Subjects also consented to the publication of the results of this research while maintaining their confidentiality.

### Multi-omics methods

Datasets generated for multi-omics profiling span nine different biospecimen sample types: whole blood, serum, plasma, peripheral blood mononuclear cells (PBMCs), extracellular vesicles and particles, dried blood spots, skin biopsies, skin swabs and capsule swabs. These samples were subject to a spectrum of multi-omic assays, including WGS, clonal haematopoiesis, direct RNA sequencing, single-nuclei RNA sequencing, single-nuclei ATAC-seq, single-cell B cell repertoire and T cell repertoire V(D)J sequencing, proteomics, metabolomics, cell-free DNA sequencing, metagenomics and metatranscriptomics. Additional biomarkers were quantified using a CLIA lab for complete blood count, comprehensive metabolic panel and cytokine panel. These data types were generated across ten time points in total: three pre-flight (L − 92, L − 44, L − 3), three in-flight (FD1, FD2, FD3), one post-flight (R + 1) and three recovery (R + 45, R + 82, R + 194).

Multi-omic feature counts describe the datasets published in refs. ^4,5^. Transcripts detected in whole blood RNA sequencing were assembled using StringTie^52^. m6A modifications were quantified using m6Anet^53^ with a probability threshold of 0.9. Genes detected from skin biopsy had a count of at least five from the normalized count matrix from the NanoString GeoMx NGS DnD Pipeline. The number of ATAC-seq peaks and genes detected from the single-nuclei data was quantified using the cellranger-arc (v2.0.0) count algorithm from 10x Genomics. We followed the 10x single-cell multi-ome analysis pipeline as previously reported^48^ and adapted for this data as described here. Shotgun metagenomic and metatranscriptomic sequencing reads were deduplicated, filtered for human sequences via alignment to the human reference genome (Hg38) and trimmed for adaptor contamination. Fungal, viral and bacterial taxonomic composition was computed via masked read alignment to a database containing all complete genomes in RefSeq using kraken2 (confidence = 0.2)^54^. For assembly-based approaches, quality-controlled (unmasked) reads were assembled with MetaSPAdes^55^. Bins were generated with MetaBAT2 (ref. ^56^), and Open-Reading-Frames were identified with bakta^57^ and clustered into a non-redundant gene catalogue with mmseqs2 (ref. ^58^). Assembled viral genomes were identified among contigs with CheckV^59^. Additional details can be found in ref. ^10^.

### Virome methods

Highly multiplexed, epitope-resolved IgG reactivity analysis across the human virome was performed on reconstituted dried blood samples using DNA-barcoded peptide (PepSeq) assays. The HV2 library has been previously described^15^ and consists of 15,000 30mer peptides covering 80 viral species and selected based on earlier evidence of reactivity in other cohorts. PepSeq libraries were synthesized and used to profile IgG binding, as previously described^16,17^. Briefly, DNA-barcoded peptide libraries were generated using bulk in vitro enzymatic reactions, starting with the PCR amplification of oligonucleotide templates and their transcription to generate mRNA. The product was ligated to a hairpin oligonucleotide adaptor bearing a puromycin molecule tethered by a polyethylene glycol spacer and used as a template in an in vitro translation reaction. Finally, a reverse transcription reaction, primed by the adaptor hairpin, was used to generate cDNA, and the original mRNA was removed using RNAse. To perform serological assays, 0.1 pmol of the resulting DNA-barcoded peptide library (5 μl) was added to 5 μl of neat, reconstituted blood spot solution and incubated overnight. The binding reaction was applied to prewashed protein G-bearing beads, washed, eluted and indexed using barcoded DNA oligos. Following PCR cleanup, products were pooled, quantified and sequenced using an Illumina NextSeq instrument yielding a depth of more than 900,000 reads per sample.

*Z*-score enrichment values for each peptide in each sample were generated from raw sequence reads in a two-step process using PepSIRF v.1.4.0, an open-source software package for the analysis of highly multiplexed serology^60^ data. First, reads were demultiplexed and mapped to members of the HV2 library using the demux module to generate integer count values for each sample peptide. Next, peptides with similar abundances in the buffer-only negative control samples were grouped into bins and used to generate *z*-scores for each data point, representing the distance (in standard deviations) of each data point from its unenriched distribution mean. log_2_-transformed offset-adjusted *z*-scores (log_2_(*z* + 8) − 3) were used for downstream analyses. At an adjusted *z*-score threshold of 0.75, we detected 45 virus species (Supplementary Table 3) for which at least 1 peptide was reactive in at least 1 sample. To identify viral events, we applied peptide set enrichment analysis to all pairs of consecutive samples as previously described^19^, and used a *P* value threshold of 1 × 10^−5^.

### Membrane printing

0*g*-VFI membranes were fabricated using nitrocellulose membrane sheets (9 × 8 cm), which were prepared into target membranes on a bench-top CO_2_ laser cutter at 1% power, 100% speed and 3 mm depth. These target membranes have circular discs (6 mm diameter) cut into them in a 6 × 5 design with fiducial markers for targeting the dispensing locations during microarray printing. Control antibody reagent (mouse IgG), rabbit anti-human CRP (11250-R106, SinoBiological) and goat anti-human IgM (109-005-129, Jackson ImmunoResearch) capture antibodies were diluted to working concentrations (0.5 mg ml^−^^1^) using filtered 1× PBS. In a clean-room environment, a Nano-Plotter NP2.1 was used for non-contact piezoelectric microarray dispensing of capture antibodies onto the circular discs of a target nitrocellulose membrane (0.2 µm pore size). To prevent evaporation of reagents during printing, an ambient humidity of 55% was maintained using a humidifier. The nozzle hydrostatic pressure was set with the water level of the pressure compensation vessel at the pipette tip height. Spot-front-end software was utilized to co-ordinate a spotting plan for use in the Nano-Plotter Controller software (NPC16) to dispense a nine-spot pattern (350 µm period) of the antibody microarray onto the centre of each circular disc. A 384-well microplate was used to aspirate the antibodies during dispensing. The test reagents were dispensed in 20 droplets per spot at their respective working concentrations at the top two rows (three spots per row for each reagent) of the nine-spot pattern. Ten droplets of the control reagent were dispensed at the bottom three spots of the nine-spot pattern. The immunoassay membranes were then stored in aluminium pouches with silica bead desiccant for later use.

### Preparation of conjugation pad

Conjugate pads were fabricated using polyester fibre with binder (Grade 6614, Ahlstrom Munksjo), which were cut into circular discs (10.5 mm) on a bench-top CO_2_ laser cutter. Gold nanoparticles conjugated anti-rabbit IgG (kindly provided by Dr AuCoin at the University of Nevada-Reno) were incubated with mouse anti-human CRP (11250-R106, SinoBiological) and IgM (MA5-14729, ThermoFisher) for 10 min. The conjugate mix was then dispensed and dried on the conjugate pads at 25 °C for 2 h.

### Assembly of vertical flow apparatus

The 0*g*-VFI consists of a stacked multilayer of pads and membranes assembled in 3D-printed plastic caps and capsules (Extended Data Fig. 3a). The sandwich immunoassay on the 0*g*-VFI is performed at the multiplexed sensing nitrocellulose membrane, which contains the nine immunoreaction spots. Above the 0*g*-VFI membrane are functional paper layers, which decrease flow surface area to achieve uniform vertical flow (flow-directing pad), decrease flow rate to increase intensity (retarding pad), generate the assay colour (conjugation pad) and collect plasma samples (sample pad). These layers are all contained in a 3D-printed pad holder, placed in a membrane housing that allows contact between the 0*g*-VFI membrane and cotton absorbing pads. The user-friendly device also contains capsules within which are wet pads (assay or washing buffer) that trigger fluid transfer once screwed to the membrane housing and placed in contact with the absorbing pads. In total, the 0*g*-VFI contains four modules consisting of the membrane housing protected with a cap, assay buffer capsule, washing buffer capsule and magnifier cap, all of which fit in small zip bags (Extended Data Fig. 3b). Overall, the assay takes up to 20 min due to its simple assembly sequence using the screw cap and reservoir design of the 0*g*-VFI platform (Extended Data Fig. 3c). A user manual and instruction video were made to detail the workflow procedure to SpaceX personnel and Inspiration4 crew. The user manual was integrated in the 0*g*-VFI kit and was thus available in-flight. The Inspiration4 crew were trained by SpaceX personnel and were instructed to work in pairs for sample collection and to self-perform the 0*g*-VFI procedure.

All plastic components of the 0*g*-VFI were designed in SolidWorks and 3D-printed in house at the Center for Applied NanoBioscience and Medicine using a Surgical Guide Resin material from Formlabs. Using the Formlabs 3B stereolithography printer, the 0*g*-VFI was printed and then washed for 20 min in 99% isopropyl alcohol to clear loose and uncured resin. Curing was then performed under intense ultraviolet light at 70 °C for 30 min, followed by trimming of the support scaffolds. First, the different pad layers were stacked inside of the pad holder as follows (ordered top to bottom): sample pad (made from chopped glass with binder (Grade 8950, Ahlstrom Munksjo)), polyethersulfone filter, conjugate pad, retarding and flow-directing pads both made of thin glass fibres (Grade 8950, Ahlstrom Munksjo). All layers were assembled with double-sided, medical-grade pressure-sensitive adhesive (ARcare 90106NB). Second, a 3.5-cm-diameter cotton pad (Grade 320, Ahlstrom Munksjo) was positioned in the membrane housing with a 6-mm-diameter cotton pad (Grade 222, Ahlstrom Munksjo) on top, both acting as absorbent pads. The 0*g*-VFI printed membrane was then placed on top of the absorbing pads and 400 µl of blocking buffer (10 mM borate buffer (pH 8) with 2.5% Triton X-100, 1% bovine serum albumin, 0.2% polyvinylpyrrolidone-40 and 0.1% sucrose filtered through a 0.2 µm polyethersulfone filter membrane) was pipetted on it and incubated for 30 min. Then, the pad holder was positioned inside the housing membrane and the device was tightly closed with a lid. Separately, wet pads saturated with a TritonX100 (0.1%), albumin (0.5%), PBS (0.1 M) solution and a 50 mM carbonate/bicarbonate buffer (washing buffer) were placed in the assay buffer pad capsule and the washing buffer pad capsule, respectively. Plasma was separated from fingerstick-collected whole blood using a modified A-PON plasma separator cartridge (GattaCo). As drawing blood using conventional methods is difficult in spaceflight for novice astronauts, whole blood was collected via fingerstick using a Food and Drug Administration-approved lancet to promote successful sample collection by the Inspiration4 crew.

### Ultrasound methods

All imaging was performed with a Butterfly iQ+ handheld ultrasound system (Butterfly Network) with the Butterfly mobile app running on iPhone12 (Apple, Inc.). The device was used in approved preset modes for each procedure (ophthalmic, vascular and bladder). Eighty-nine imaging instances (multiframe cine of varied length) were collected pre-flight and 108 (7.68 GB) images were collected in-flight. All in-flight data (27 instances per astronaut on average; range = 18–32) were stored on the local device (iPhone 12) until secure transfer to a DICOM (Digital Imaging and Communications in Medicine) server upon return to Earth. Image analysis was performed in Osirix MD DICOM software (Pixmeo). Of the *n* = 106 in-flight imaging instances analysed, *n* = 73 were related to IJV, *n* = 14 to urinary bladder and *n* = 19 were ophthalmic.

All astronauts were self-scanning operators. All pre-flight crew interactions (experiment briefing, familiarization and training, and baseline data collection) were conducted by SpaceX personnel using limited time allocations. Using a previously untested approach to experiment execution, the investigative team relied on experiment-specific JIT instructions to communicate both conceptual and procedural information to SpaceX personnel (for baseline data collection and familiarization) and to the Inspiration4 crew (for autonomous in-flight data collection). In-flight imaging data were obtained via self-scanning and used potable water as a coupling medium in lieu of ultrasound gel. Bladder scans were collected both pre- and post-void, whenever possible. IJV scans were performed with and without inspiratory resistance generated by an ITD (ResQGARD ITD7, Zoll).

Given the risks to data quality posed by limitations of imaging expertise, and to evaluate the effectiveness of the JIT tools, a formalized data quality assessment was conducted in the initial phase of data analysis. This included the scoring of each imaging instance for anatomical accuracy and technical quality on a scale of 0–3 (0 = no useful information, 3 = clinical quality). Anatomical accuracy criteria included whether the target was clearly and contiguously visible, along with essential anatomical landmarks; technical quality criteria included elements such as image clarity, gain, acoustical interference, artefact and shadowing. An overall success score was derived as the mean of these two scores, with a usability threshold set at 2.0. This quality filter served to reject anatomically inaccurate or technically flawed images. To determine whether the success of imaging instances varied by anatomical target, a one-way ANOVA tested for in-flight differences in imaging success scores among anatomical targets (that is, bladder, IJV and eye); post hoc pairwise comparisons between anatomical targets were conducted via Tukey’s honest significant difference. To evaluate the effect of impedance breathing on IJV CSA, a series of paired, two-tailed Student’s *t*-tests were conducted; differences between normal and impedance breathing were evaluated by averaging IJV CSA for each condition within each mission phase (that is, pre-flight and in-flight).

### Ocular misalignment methods

The otolith organs of the vestibular system transduce linear acceleration and gravity. The sense organs consist of a mass of calcium carbonate crystals (otoconia) that overlie a membrane that is innervated with hair cells. Motion of the crystals relative to the hair cells bends the hair cells, and this bending modifies their firing rates and so provides information on linear acceleration to the central vestibular system. While generally symmetric, there is reason to believe that there are slight asymmetries between the otolith organs on the two sides of the head, in otoconial mass or synaptic sensitivity. This asymmetry is compensated by central neural processes^27^, but this compensation becomes inappropriate in gravity fields other than 1*g*, leading to changes in vertical and torsional ocular alignment^61,62^. In particular, the magnitude of torsional misalignment in altered *g* levels has been associated with susceptibility to SMS^40,41,63^. *G*-dependent changes in vertical alignment have also been demonstrated in parabolic flight and laboratory studies^62,64^. Data on SMS in-flight were obtained via self-report.

A perceptual-nulling technique was used to measure misalignment between the eyes (skew) in both vertical and torsional directions^65^. For these tests, the subject views a red line and a blue line on the touchscreen of a tablet computer, through colour-matched red and blue filters, one over each eye. This provides independent images to each eye. Since the test is performed in darkness, there is no visual information that is seen by both eyes together, and hence there are no visual cues to align the eyes (which would fuse the images on the two retinas). One line remains fixed on the screen, while the other line is positioned by the subject, either vertically or torsionally. The subject’s goal is to adjust one line until it appears to be aligned with the other, stationary, line (that is, to null any apparent vertical or rotational offset between the lines). The final amount by which the lines are separated from one another vertically or rotated relative to one another provides a measure of vertical or torsional ocular misalignment, respectively, which produces measures of VAN and TAN. Astronauts completed VAN and TAN measures twice pre-flight and twice post-flight, where each session consisted of 11 VAN and 11 TAN trials. There was no in-flight testing.

Statistical testing to evaluate associations of torsional (TAN) and vertical (VAN) ocular misalignment with SMS were carried out via a series of two-sample *t*-tests within each astronaut individually to provide information on the following: (1) whether the two pre-flight datasets are the same or different from each other, (2) whether the two post-flight datasets are the same or different from each other and (3) whether the (grouped) pre-flight data are different from the (grouped) post-flight data. The *t*-tests were performed after removing outliers, which were defined as values more than three scaled median absolute deviations from the median, where median absolute deviation is a measure of the deviation of the values from the median (analogous to the standard deviation for data from normal distributions) and can be reduced to a sequential decision tree for prediction of individual SMS susceptibility (Supplementary Note 2). There are several reasons for this individual testing as opposed to a pooled analysis across subjects. First, the high variability across subjects (typical of many neurovestibular assessments), combined with the small subject pool, makes it unlikely that there would be sufficient statistical power in a pooled analysis. Second, individual testing permits a stepwise approach in which a series of tests can lead to a predictive metric. Finally, SMS is notoriously idiosyncratic, and spaceflight measures are subject to many confounds, which supports a focused longitudinal examination.

### Cardiovascular function

The Apple Watch Series 6 was used to objectively measure cardiovascular function, which was indexed by blood oxygen saturation levels, heart rate and HRV, as well as activity and energy consumption. The Apple Watch also measured sound pressure levels. The crew donned the Apple Watch for selected periods in all three phases of the mission, including pre-flight, in-flight and post-flight (Extended Data Fig. 1). The crew donned the Apple Watch from L − 22 to L − 20 and had an average wear time of 2.7 ± 0.3 (s.d.) days (range = 2.4–3.0 days), in-flight from FD2 to FD3 with an average wear time of 1.2 ± 0.1 (s.d.) days (range = 1.2–1.4 days) and post-flight from R + 0 to R + 10 with an average wear time of 5.8 ± 3.4 (s.d.) days (range = 2.2–9.1 days).

### Neurocognitive functioning

Neurocognitive functioning was assessed using NASA’s cognition test battery^33^, which consists of 10 brief cognitive tests that probe a range of neurocognitive domains relevant to the challenges of spaceflight and includes the psychomotor vigilance test, matrix reasoning test, abstract matching, line orientation test, visual object learning test, motor praxis task, emotion recognition task, digit–symbol substitution task, fractal 2-back and balloon analogue risk test. Developed for high-performing astronauts, cognition has been deployed in long-duration spaceflight studies^1^, as well as in ground-based studies that simulate aspects of spaceflight in analogue environments^66–68^. Cognition was administered with the Joggle Research App (Pulsar Informatics, Inc.) on an iPad Mini Series 4 twice pre-flight (L − 47 and L − 22), up to three times in-flight and twice post-flight (R + 0 and R + 1). Cognition data were corrected for practice and stimulus set effects after applying another correction for the fact that tests were performed on an iPad instead of a laptop^69,70^. After these corrections, the expectation is that test results do not change with repeated administration.

### Alertness and mood survey

Before performing each cognition test battery, astronauts reported on their behavioural state using the AMS^50^ via the Qualtrics application on the Apple iPad Mini Series 4. Developed for astronauts, the 18-item AMS measures behavioural responses to the challenges of both long- and short-duration spaceflight. Fourteen of the 18 AMS items were surveyed in the Inspiration4 crew to reduce time burden, and the four items that were removed had significant overlap with other AMS items. Ten AMS items (monotony, boredom, depression, stress, physical exhaustion, sickness, unhappiness, sleepiness, workload and sleep quality) prompted astronauts to rate each item using 11-point Likert scales (range = 0–10). The remaining AMS items assessed astronaut sleep timing and duration, crew conflict and medication use (stimulant/depressant).

### Statistical analysis of cognition, AMS and Apple watch data

All statistical analyses were conducted using SAS v.9.4. Before statistical analysis of cognitive performance, all cognition data were standardized via *z*-scoring to facilitate comparisons across the different neurocognitive domains, as well as to allow the effects to be interpreted as effect sizes. Mixed-effects models using PROC MIXED with a random subject intercept were used to test astronaut behavioural and physiological responses to short-duration spaceflight across mission phases. Repeated measures were nested within subjects and differences between responses during both the in-flight and post-flight periods were tested relative to the pre-flight period using change scores generated for each astronaut from the average of an outcome for each mission phase. Post hoc analyses that tested differences between astronauts and mission phases were corrected for multiple comparisons based on the false discovery rate method with a significance threshold of *P* < 0.05 (refs. ^51^); both unadjusted and adjusted *P* values are reported. For crew-level differences between mission phases, Type III effects are reported.

### Reporting summary

Further information on research design is available in the Nature Portfolio Reporting Summary linked to this article.

## Online content

Any methods, additional references, Nature Portfolio reporting summaries, source data, extended data, supplementary information, acknowledgements, peer review information, details of author contributions and competing interests, and statements of data and code availability are available at 10.1038/s41586-024-07648-x.

### Supplementary information

Supplementary Information The Supplementary Information contains Supplementary Notes 1–3 and Tables 1–7. The notes provide information on and results of statistical analyses. The tables provide information on the amount and type of data collected, as well as findings from immunological, cardiovascular and neurobehavioural studies of the Inspiration4 astronauts.

Reporting Summary

## Data Availability

Datasets have been uploaded to four different data repositories: the NASA Open Science Data Repositories (osdr.nasa.gov; comprising GeneLab^71^ and the Ames Life Sciences Data Archive (ALSDA)^2,72^, the SOMA Data Portal and the TRISH EXPAND TrialX database).
