# Supplementary material for: Molecular and physiological changes in the SpaceX Inspiration4 civilian crew
Source: Nature. 2024 Jun 11;632(8027):1155–64. doi: 10.1038/s41586-024-07648-x (PMC11357997; doi:10.1038/s41586-024-07648-x)
Supplement: Supplementary file 1 — The Supplementary Information contains Supplementary Notes 1–3 and Tables 1–7. The notes provide information on and results of statistical analyses. The tables provide information on the amount and type of data collected, as well as findings from immunological, cardiovascular and neurobehavioural studies of the Inspiration4 astronauts. [file 41586_2024_7648_MOESM1_ESM.docx]

**Molecular and physiologic changes in the SpaceX Inspiration4 civilian crew**

Authors:

Jones, C.W.^1*^, Overbey, E. G.^2,3,4*^, Lacombe, J.^5,6*^, Ecker, A.J.^1^, Meydan, C.^2,3,4^., Ryon, K.^2,3,4^, Tierney, B.^2,3,4^, Damle, N.^2,3,4^, MacKay, M.^2,3,4^, Afshin, E.E.^2,3,4^, Foox, J.^2,3,4^, Park, J.^2,3^, Nelson, T.^7^, Suhail, M.^8^, Byhaqui, S.G.^8^, Aslam, B.^8^, Tali, U.A.^8^, Nisa, L.^8^, Menon, P.^8^, Patel, C.O.^8^, Khan, S.A.^8^, Ebert, D.J.^9^, Everson, A.^9^, Schubert, M.C.^10^, Ali, N.N.^10^, Sarma, M.S.^10^, Kim, J.^2,3^, Houerbi, N.^2,3,^, Grigorev, K.^2,3,^, Garcia Medina, S.^2,3^, Summers, A.J.^5^, Gu, J.^5,6^, Altin, J.A.^11^, Fattahi, A.^5^, Hirzallah, M.I.^12,13^, Wu, J.H.^13,14^, Stahn, A.C.^1^, Beheshti, A.^15,16^, Klotz, R.^17^, Ortiz, V.^17^, Yu, M.^17^, Patras, L.^18,19^, Matei, I.^18,20^, Lyden, D.^18,20^, Melnick, A.^2^, Banerjee, N.^21^, Mullane, S.^21^, Kleinman, A.^2,3^, Loesche, M.^21^, Menon, A.S.^22^, Donoviel, D.B.^13,14,+^, Urquieta, E.^13,14,+^, Mateus, J.^21+^, Sargsyan, A.E.^9+^, Shelhamer, M.^10+^, Zenhausern, F.^5,6,11,23+^, Bershad, E.M.^13,14+^, Basner, M.^1+^ , Mason, C.E.^2,3,4+^

*Co-First Authors

^+^Co-Senior Authors

Corresponding authors: chm2042@med.cornell.edu (Christopher E. Mason); basner@pennmediicne.upenn.edu (Mathias Basner)

**Supplementary Information**

**Table of Contents**

[Supplementary Note 1. Statistical considerations of sample size 3](#_Toc166450841)

[Supplementary Note 2. Statistical analysis of otolith asymmetry and space motion sickness 4](#_Toc166450842)

[Supplementary Note 3. Astronaut cognitive performance in-flight relative to pre-flight 5](#_Toc166450843)

[Supplementary Table 1: Total observations by data types. 6](#_Toc166450844)

[Supplementary Table 2: Biospecimens, associated assays, and Open Science Data Repository (OSDR) identifiers. 7](#_Toc166450845)

[Supplementary Table 3: Virus species with IgG reactivity in ≥1 sample. 8](#_Toc166450846)

[Supplementary Table 4: Statistical significance from testing astronaut profiles of vertical ocular misalignment (Vertical Alignment Nulling) across mission phases. 10](#_Toc166450847)

[Supplementary Table 5: Statistical analysis of astronaut cardiovascular physiology and spacecraft environmental measures across mission phases. 11](#_Toc166450848)

[Supplementary Table 6: Statistical analysis of astronaut performance on the 10 Cognition tests across mission phases. 12](#_Toc166450849)

[Supplementary Table 7: Statistical analysis of astronaut ratings of behavioral states on the Alertness of Mood Survey across mission phases. 13](#_Toc166450850)

[Supplementary References 14](#_Toc166450851)

**Supplementary Note 1. Statistical considerations of sample size**

The largest problem with most human spaceflight studies is the limited sample size, but greater power can come from continued, longitudinal sampling. To address the challenges of limited samples size, several methods were examined: (1) generalized linear models (GLM) that calculate changes as a function of time, (2) local and global convolution methods^1^, and (3) established techniques for modeling longitudinal statistics^2^. This includes estimates for (1) comparison of two groups at a single time point, as well as (2) comparison of two groups across time, as well as comparisons to other cohorts’ data (e.g., NASA Twins Study^2^) whenever applicable. They can be modeled as such:


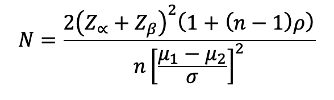


Where Zα is the value of the standardized score cutting off α/2 proportion of each tail of a standard normal distribution (for a two-tailed hypothesis test), and Zβ is the value of the standardized score cutting off the upper β proportion, σ2 is the assumed common variance in the two groups, and (μ1−μ2) is the difference in means of the two groups for a genomic, epigenomic, and transcriptional measure, n is the number of time points and ρ is the correlation of n repeated measurements. Based on the Hedeker calculations and given the samples in our study, we have 90% power at 1% FDR, with a ρ=0.8 to see an effect size of 1.8. Most thresholds used in this paper are at a fold change of >2, to be conservative. We also checked for normality in all our data sets before comparisons and used non-parametric methods (e.g., Fisher’s Exact Test) when normality was absent or unclear.

**Supplementary Note 2. Statistical analysis of otolith asymmetry and space motion sickness**

To evaluate patterns of ocular misalignment as they related to space motion sickness (SMS), we carried out two-sample *t*-tests to verify the pattern seen in **Fig. 4**. These *t*-tests were performed for each subject individually to provide information on the following:

- Whether the two pre-flight datasets are the same or different from each other?
- Whether the two post-flight datasets are the same or different from each other?
- Whether the (grouped) pre-flight data are different from the (grouped) post-flight data?

The *t*-tests were performed after removing outliers, which were defined as values more than three scaled median absolute deviations (MAD) from the median, where MAD is a measure of the deviation of the values from the median (analogous to the standard deviation for data from normal distributions). Results of these statistical tests are presented in Supplementary Table 4, and can be reduced to a sequential decision tree for prediction of individual SMS susceptibility:

- Are the pre-flight and post-flight data different from each other?
  - If no, then SMS is indicated (no adaptation to spaceflight has occurred, and there is no change in ocular alignment).
  - If yes, are the two pre-flight and the two postflight datasets the same?
    - If no, then SMS is indicated. There is not consistent compensation for otolith asymmetry, as reflected in ocular alignment.
    - If yes, then no SMS is indicated. There is consistent compensation for asymmetry, which is different between pre-flight and post-flight.

**Supplementary Note 3. Astronaut cognitive performance in-flight relative to pre-flight**

Astronaut cognitive performance exhibited significant differences in-flight relative to pre-flight (Fig. 5a and Supplementary Table 6), which was primarily in response speed on Cognition assays. Slower response speed in-flight relative to pre-flight was observed on the Psychomotor Vigilance Test (PVT; β=-1.041, p=0.0013; adjusted P<0.01), Digit Symbol Substitution Test (DSST; β=-1.431, P<0.0001; adjusted P<0.0001), Fractal 2-Back (F2B; β=-1.286, p=0.0017; adjusted P<0.01), and Motor Praxis (MP; β=-1.068, p=0.0202; adjusted P<0.05), while faster response speed was observed for both the Balloon Analog Risk Task (BART; β=1.118, p=0.0038; adjusted P<0.01) and Abstract Matching (AM; β=1.040, p=0.0015; adjusted P<0.01); this faster response speed in-flight was driven by slower response speeds pre-flight as astronauts were likely responding more deliberately as they learned the BART and AM tasks. The faster AM response speed in-flight was accompanied by lower AM accuracy (β=-0.854, p=0.0069; adjusted P<0.05) and the slower MP response speed in-flight was paired with lower MP accuracy (β=-1.378, p=0.0022; adjusted P<0.05); significant changes in accuracy did not accompany changes in response speed in-flight relative to pre-flight for the PVT (β=-0.684, p=0.13), DSST (β=-0.674, p=0.10), F2B (β=0.152, p=0.70), or risk taking on the BART (β=-0.312, p=0.33). Consistent with prior studies of astronauts conducting both short- and long-duration spaceflight missions, not all cognitive domains were affected by spaceflight2,26. Astronaut cognitive performance was not significantly different in-flight relative to pre-flight for either the speed or accuracy outcomes on the Visual Object Learning Test (VOLT; response speed: β=-0.227, p=0.55; accuracy: β=0.291, p=0.52), Line Orientation Test (LOT; response speed: β=0.266, p=0.57; accuracy: β=-0.166, p=0.70), Emotion Recognition Task (ERT; response speed: β=0.820, p=0.037; adjusted P>0.05; accuracy: β=-0.289, p=0.45), or the Matrix Reasoning Task (MRT; response speed: β=0.875, p=0.057; accuracy: β=-0.468, p=0.28).

Supplementary Table 1: Total observations by data types.

| **Data Type** | **Data Description** | **Total Data Points** |
| --- | --- | --- |
| Observations | Surveys neurocognitive performance, sensorimotor testing | 360,778 (318 surveys) |
| Ultrasound Imaging | Multi-frame image files with metadata from vascular, ophthalmic, and abdominal targets | 197 (multi-frame image files with metadata, DICOM standard) |
| Biospecimen Samples | Blood, saliva, capillary blood, venous blood, and Dragon capsule surface samples collected | 2,911 |
| Wearable | Heart rate, ECG, Oxygen saturation and other metrics from Apple Watch | 327,759 |
| Telemetry | Cabin pressure, Partial O_2_/CO_2_/N_2_ relative humidity and temperature readings | 6,621,851 |

Supplementary Table 2: Biospecimens, associated assays, and Open Science Data Repository (OSDR) identifiers.

| **Biospecimen** | **Assay(s)** | **OSDR Identifier** |
| --- | --- | --- |
| Whole Blood | - ONT Direct RNA-seq - Complete Blood Count (CBC) | OSD-569 |
| PBMC | - 10X Chromium Multiome Kit/ single-nuclei RNA-seq and ATAC-seq - 10X Chromium T-cell repertoire (TCR) and B-cell repertoire (BCR) sequencing | OSD-570 |
| Blood Plasma | - Proteomics (Seer Proteograph) - Proteomics of blood extracellular vesicles and particles - Proteomic assay of blood plasma metabolome - cfDNA sequencing from blood plasma | OSD-571 |
| Crew Swabs  (Oral, Nasal, Skin) | - Metagenomics - Metatranscriptomics | OSD-572 |
| Environmental Swabs | - Metagenomics - Metatranscriptomics | OSD-573 |
| Skin Biopsy | - Nanostring GeoMx spatial transcriptomics - Nanostring GeoMx tissue images - Deltoid swab metagenomics from biopsy site - Deltoid swab metatranscriptomics from biopsy site | OSD-574 |
| Blood Serum | - Comprehensive Metabolic Panel - Cytokine/Chemokine Biomarker Panel - Cardiovascular Biomarker Panel | OSD-575 |

The assays used for the multi-omic profiling of the I4 crew are listed for each biospecimen type with Open Science Data Repository (OSDR) identifiers.

Supplementary Table 3: Virus species with IgG reactivity in ≥1 sample.

| **Species name** | **Abbreviation used in Fig. 2** |
| --- | --- |
| Rhinovirus A | HRV-A |
| Rhinovirus C | HRV-C |
| Rhinovirus B | HRV-B |
| Enterovirus B | EV-B |
| Enterovirus C | EV-C |
| Human gammaherpesvirus 4 | HHV-4 |
| Enterovirus A | EV-A |
| Human mastadenovirus C | HAdV-C |
| Enterovirus D | EV-D |
| Human alphaherpesvirus 1 | HHV-1 |
| Norwalk virus | NV |
| Severe acute respiratory syndrome coronavirus 2 | SARS-CoV-2 |
| Human respirovirus 3 | HRV3 |
| Human betaherpesvirus 7 | HHV-7 |
| Human betaherpesvirus 5 | HHV-5 |
| Human mastadenovirus B | HAdV-B |
| Human mastadenovirus D | HAdV-D |
| Human betaherpesvirus 6 | HHV-6 |
| Human orthopneumovirus | HOPV |
| Rotavirus A | RV-A |
| Human mastadenovirus A |  |
| Human alphaherpesvirus 2 |  |
| Influenza A virus |  |
| Human metapneumovirus |  |
| Human rubulavirus 4 |  |
| Influenza B virus |  |
| Betacoronavirus 1 |  |
| Human alphaherpesvirus 3 |  |
| Human coronavirus 229E |  |
| Alphapapillomavirus 9 |  |
| Mammalian orthoreovirus |  |
| Parechovirus A |  |
| Measles morbillivirus |  |
| Human respirovirus 1 |  |
| Rotavirus C |  |
| Human coronavirus NL63 |  |
| Rubella virus |  |
| Hepacivirus C |  |
| Sapporo virus |  |
| **Species name** | **Abbreviation used in Fig. 2** |
| Human bocavirus |  |
| Betapapillomavirus 2 |  |
| Alphapapillomavirus 7 |  |
| Betapapillomavirus 1 |  |
| Orthohepevirus A |  |
| Human polyomavirus 3 |  |

| Supplementary Table 4: Statistical significance from testing astronaut profiles of vertical ocular misalignment (Vertical Alignment Nulling) across mission phases. | | | | |
| --- | --- | --- | --- | --- |
|  | C001 | C002 (SMS) | C003 | C004 (SMS) |
| Pre-flight: two VAN ocular misalignment measures | 0.09 ns  (0.43) | **0.004 sig**  **(0.17)** | 0.46 ns  (0.33) | **0.008 sig**  **(1.95)** |
| Post-flight: two VAN ocular misalignment measures | **0.002 sig**  **(0.41)** | 0.21 ns  (0.15) | 0.45 ns  (0.17) | 0.52 ns  (0.44) |
| VAN change from pre-flight to post-flight | **<0.001 sig**  **(0.94)** | 0.68 ns  (0.03) | **<0.001 sig**  **(1.48)** | **<0.001 sig**  **(2.20)** |
| P-values (and effect sizes in parentheses) obtained from two-sample *t*-tests comparing vertical ocular misalignment (VAN) measures collected from astronauts pre-flight and post-flight. A series of *t*-tests was conducted within each astronaut to compare 1) the two VAN measures collected pre-flight (first data row), 2) the two VAN measures collected post-flight (second data row), and 3) the change in VAN measures from pre-flight to post-flight (third row). Astronauts who experienced space motion sickness (SMS) in spaceflight are identified with SMS. The statistical significance level was P<0.01 and bolded P-values denote statistical significance. ns = not a significant difference; sig = significant difference; SMS = space motion sickness. | | | | |

Supplementary Table 5: Statistical analysis of astronaut cardiovascular physiology and spacecraft environmental measures across mission phases.

|  | **Pre-flight relative to in-flight** | | | | | | **Post-flight relative to in-flight** | | | | | | **Post-flight relative to pre-flight** | | | | | |
| --- | --- | --- | --- | --- | --- | --- | --- | --- | --- | --- | --- | --- | --- | --- | --- | --- | --- | --- |
| **Apple watch metric** | **Estimate** | | **SEM** | | **P-value** | | **Estimate** | | **SEM** | | **P-value** | | **Estimate** | | **SEM** | | **P-value** | |
| Heart rate (bpm) |  |  | |  | |  | |  | |  | |  | |  | |  | |  |
| C001 | 1.140 | | 2.701 | | 0.67 | | **10.121** | | **2.637** | | **0.0001***** | | **8.981** | | **2.177** | | **<0.0001****** | |
| C002 | 4.586 | | 3.056 | | 0.13 | | **17.590** | | **3.274** | | **<0.0001****** | | **13.005** | | **3.023** | | **<0.0001****** | |
| C003 | 5.554 | | 3.336 | | 0.097 | | -0.443 | | 3.857 | | 0.91 | | -5.997 | | 3.830 | | 0.12 | |
| C004 | **22.500** | | **2.849** | | **<0.0001****** | | **27.718** | | **3.035** | | **<0.0001****** | | 5.218 | | 2.849 | | 0.068 | |
| Heart rate variability (ms) | | | | | | |  | |  | |  | |  | |  | |  | |
| C001 | -7.537 | | 7.531 | | 0.32 | | **-18.794** | | **7.380** | | **0.012*** | | -11.257 | | 7.195 | | 0.12 | |
| C002 | -1.399 | | 8.009 | | 0.86 | | -11.181 | | 8.619 | | 0.20 | | -9.782 | | 8.009 | | 0.22 | |
| C003 | 0.640 | | 7.364 | | 0.93 | | -3.803 | | 7.979 | | 0.63 | | -4.443 | | 8.687 | | 0.61 | |
| C004 | **-16.632** | | **6.436** | | **0.011*** | | **-19.542** | | **7.746** | | **0.013*** | | -2.911 | | 7.464 | | 0.70 | |
| Blood oxygen saturation (%) | | | | | | |  | |  | |  | |  | |  | |  | |
| C001 | -0.008 | | 0.005 | | 0.14 | | 0.005 | | 0.006 | | 0.38 | | **0.013** | | **0.005** | | **0.017*** | |
| C002 | . | | . | | . | | . | | . | | . | | 0.002 | | 0.007 | | 0.72 | |
| C003 | . | | . | | . | | . | | . | | . | | -0.009 | | 0.011 | | 0.42 | |
| C004 | **0.037** | | **0.006** | | **<0.0001****** | | **0.045** | | **0.007** | | **<0.0001****** | | 0.008 | | 0.007 | | 0.24 | |
| Active energy burned (cal/h) | | | | | | |  | |  | |  | |  | |  | |  | |
| C001 | **24.434** | | **7.803** | | **0.0018*** | | **21.291** | | **7.858** | | **0.007*** | | -3.143 | | 6.284 | | 0.62 | |
| C002 | 12.517 | | 8.449 | | 0.14 | | 22.696 | | 9.427 | | 0.02 | | 10.179 | | 8.782 | | 0.25 | |
| C003 | 8.833 | | 8.565 | | 0.30 | | 6.014 | | 9.679 | | 0.53 | | -2.819 | | 9.016 | | 0.75 | |
| C004 | 10.604 | | 8.341 | | 0.20 | | **23.801** | | **8.926** | | **0.0079*** | | 13.196 | | 8.130 | | 0.11 | |
| Sound pressure levels (dBA) | | | | | | |  | |  | |  | |  | |  | |  | |
| C001 | **-7.152** | | **1.760** | | **<0.0001****** | | **-5.043** | | **1.711** | | **0.0033*** | | 2.109 | | 1.415 | | 0.14 | |
| C002 | 3.280 | | 2.035 | | 0.11 | | -1.064 | | 2.165 | | 0.62 | | -4.344 | | 2.024 | | 0.032 | |
| C003 | 1.840 | | 2.231 | | 0.41 | | **-19.212** | | **2.829** | | **<0.0001****** | | **-21.052** | | **2.820** | | **<0.0001****** | |
| C004 | -1.315 | | 1.869 | | 0.48 | | **6.570** | | **1.958** | | **0.0008**** | | **7.886** | | **1.853** | | **<0.0001****** | |
| Barometric pressure (kPa) | | | | | | |  | |  | |  | |  | |  | |  | |
| C001 | 0.523 | | 0.320 | | 0.10 | | **1.177** | | **0.336** | | **0.0006**** | | 0.654 | | 0.320 | | 0.042 | |
| C002 | . | | . | | . | | . | | . | | . | | 0.413 | | 0.388 | | 0.29 | |
| C003 | . | | . | | . | | . | | . | | . | | 0.578 | | 0.631 | | 0.36 | |
| C004 | 0.254 | | 0.337 | | 0.45 | | **1.388** | | **0.418** | | **0.0011**** | | **1.134** | | **0.405** | | **0.0057*** | |

Differences in individual astronaut cardiovascular physiology and exposure to spacecraft environmental characteristics, measured by the Apple watch, between mission phases were evaluated using mixed-effects models. P-values are corrected for multiple comparisons using the false discovery rate method^60^: *P<0.05; **P<0.01; ***P<0.001; ****P<0.0001. Bolded estimates, SEM and P-values denote a significant difference between mission phases. SEM = standard error; bpm = beats per minute; ms = mean seconds;

Supplementary Table 6: Statistical analysis of astronaut performance on the 10 Cognition tests across mission phases.

|  | **In-flight relative to pre-flight** | | | | **Post-flight relative to pre-flight** | | | |
| --- | --- | --- | --- | --- | --- | --- | --- | --- |
| **Cognition metric** | **Estimate** | **95% CI LOW** | **95% CI HIGH** | **P-value** | **Estimate** | **95% CI LOW** | **95% CI HIGH** | **P-value** |
| **MP Speed** | **-1.068** | **-1.954** | **-0.182** | **0.020*** | -0.132 | -1.066 | 0.802 | 0.77 |
| **MP Accuracy** | **-1.378** | **-2.207** | **-0.548** | **0.0022*** | -0.608 | -1.482 | 0.266 | 0.16 |
|  |  |  |  |  |  |  |  |  |
| VOLT Speed | -0.227 | -0.999 | 0.544 | 0.55 | 0.219 | -0.592 | 1.030 | 0.58 |
| VOLT Accuracy | 0.291 | -0.645 | 1.227 | 0.52 | 0.044 | -0.940 | 1.029 | 0.93 |
|  |  |  |  |  |  |  |  |  |
| **F2B Speed** | **-1.286** | **-2.027** | **-0.544** | **0.0017**** | -0.491 | -1.270 | 0.288 | 0.20 |
| F2B Accuracy | 0.152 | -0.665 | 0.970 | 0.70 | 0.147 | -0.713 | 1.007 | 0.73 |
|  |  |  |  |  |  |  |  |  |
| **AM Speed** | **1.040** | **0.450** | **1.629** | **0.0015**** | **1.165** | **0.546** | **1.784** | **0.0008**** |
| **AM Accuracy** | **-0.854** | **-1.445** | **-0.263** | **0.0069*** | -0.800 | -1.421 | -0.179 | 0.014 |
|  |  |  |  |  |  |  |  |  |
| LOT Speed | 0.266 | -0.683 | 1.214 | 0.57 | 0.355 | -0.643 | 1.353 | 0.47 |
| LOT Accuracy | -0.166 | -1.048 | 0.716 | 0.70 | -0.951 | -1.879 | -0.022 | 0.045 |
|  |  |  |  |  |  |  |  |  |
| ERT Speed | 0.820 | 0.055 | 1.584 | 0.037 | **1.253** | **0.448** | **2.057** | **0.004*** |
| ERT Accuracy | -0.289 | -1.076 | 0.497 | 0.45 | 0.856 | 0.028 | 1.684 | 0.043 |
|  |  |  |  |  |  |  |  |  |
| MRT Speed | 0.875 | -0.028 | 1.778 | 0.057 | **1.104** | **0.152** | **2.056** | **0.025*** |
| MRT Accuracy | -0.468 | -1.348 | 0.411 | 0.28 | -0.633 | -1.559 | 0.292 | 0.17 |
|  |  |  |  |  |  |  |  |  |
| **DSST Speed** | **-1.431** | **-1.895** | **-0.968** | **<0.0001****** | **-0.887** | **-1.374** | **-0.400** | **0.0011**** |
| DSST Accuracy | -0.674 | -1.496 | 0.148 | 0.10 | -0.129 | -0.994 | 0.735 | 0.76 |
|  |  |  |  |  |  |  |  |  |
| **BART Speed** | **1.118** | **0.407** | **1.830** | **0.0038**** | **1.123** | **0.375** | **1.871** | **0.0053*** |
| BART Risk Taking | -0.312 | -0.962 | 0.339 | 0.33 | -0.211 | -0.894 | 0.473 | 0.53 |
|  |  |  |  |  |  |  |  |  |
| **PVT Speed** | **-1.041** | **-1.623** | **-0.460** | **0.0013**** | -0.108 | -0.719 | 0.503 | 0.72 |
| PVT Accuracy | -0.684 | -1.585 | 0.217 | 0.13 | 0.036 | -0.912 | 0.984 | 0.94 |
|  |  |  |  |  |  |  |  |  |
| ALL Speed | -0.088 | -0.400 | 0.224 | 0.56 | 0.360 | 0.032 | 0.688 | 0.033 |
| ALL Accuracy | -0.456 | -0.829 | -0.083 | 0.019 | -0.226 | -0.619 | 0.166 | 0.24 |

Differences in Cognition performance (response speed and accuracy) were evaluated using mixed-effects models. P-values are corrected for multiple comparisons using the false discovery rate method^60^: *P<0.05; **P<0.01; ***P<0.001; ****P<0.0001. Bolded text and P-values denote a significant difference between mission phases. PVT = Psychomotor Vigilance Test; MRT = Matrix Reasoning Test; AM = Abstract Matching; LOT = Line Orientation Test; VOLT = Visual Object Learning Test; MP = Motor Praxis Task; ERT = Emotion Recognition Test; DSST = Digit-Symbol Substitution Task; F2B = Fractal 2-Back; BART = Balloon Analog Risk Test (BART); ALL = Aggregate Cognition performance (BART risk taking does not contribute to the accuracy score across tests).

Supplementary Table 7: Statistical analysis of astronaut ratings of behavioral states on the Alertness of Mood Survey across mission phases.

|  | **In-flight relative to pre-flight** | | | | **Post-flight relative to pre-flight** | | | |
| --- | --- | --- | --- | --- | --- | --- | --- | --- |
| **AMS metric** | **Estimate** | **95% CI LOW** | **95% CI HIGH** | **P-value** | **Estimate** | **95% CI LOW** | **95% CI HIGH** | **P-value** |
| Sleep Quality | -0.425 | -2.402 | 1.552 | 0.66 | -1.250 | -3.334 | 0.834 | 0.23 |
| High Workload | 1.549 | -0.592 | 3.690 | 0.15 | 1.500 | -0.753 | 3.753 | 0.18 |
| Sleepiness | -1.302 | -3.147 | 0.543 | 0.16 | -1.875 | -3.815 | 0.065 | 0.057 |
| **Unhappiness** | -1.099 | -2.581 | 0.382 | 0.14 | **-2.250** | **-3.812** | **-0.688** | **0.007*** |
| Sickness | 1.066 | -0.640 | 2.771 | 0.21 | 1.125 | -0.671 | 2.921 | 0.21 |
| Physical Exhaustion | -0.697 | -2.578 | 1.185 | 0.45 | -1.250 | -3.228 | 0.728 | 0.20 |
| Stress | -0.953 | -3.078 | 1.172 | 0.36 | -2.625 | -4.866 | -0.384 | 0.024 |
| Depression | -0.416 | -0.970 | 0.138 | 0.13 | -0.625 | -1.209 | -0.041 | 0.037 |
| **Boredom** | -1.291 | -2.476 | -0.105 | 0.034 | **-2.125** | **-3.374** | **-0.876** | **0.002*** |
| Monotony | 0.164 | -0.945 | 1.272 | 0.76 | -0.125 | -1.292 | 1.042 | 0.83 |

Differences in astronaut report of behavioral states using the Alertness and Mood Survey^59^ were evaluated using mixed-effects models. P-values are corrected for multiple comparisons using the false discovery rate method^60^: *P<0.05; **P<0.01; ***P<0.001; ****P<0.0001. Bolded text and P-values denote a significant difference between mission phases.

Supplementary References

1 Hooper, S. D. *et al.* Identification of tightly regulated groups of genes during *Drosophila melanogaster* embryogenesis. *Mol. Syst. Biol.* **3**, 72 (2007).

2 Hedeker, D., Gibbons, R. D. & Waternaux, C. Sample size estimation for longitudinal designs with attrition: Comparing time-related contrasts between two groups. *J. Educ. Behav. Stat.* **24**, 70-93 (1999).
