# Supplementary material for: Molecular and physiological changes in the SpaceX Inspiration4 civilian crew
Source: Nature. 2024 Jun 11;632(8027):1155–64. doi: 10.1038/s41586-024-07648-x (PMC11357997; doi:10.1038/s41586-024-07648-x)
Supplement: Supplementary file 2 — Reporting Summary [file 41586_2024_7648_MOESM2_ESM.pdf]

nature portfolio

Corresponding author(s): Mathias Basner, Christopher E. Mason

Last updated by author(s): Nov 23, 2023

Reporting Summary

Nature Portfolio wishes to improve the reproducibility of the work that we publish. This form provides structure for consistency and transparency in reporting. For further information on Nature Portfolio policies, see our Editorial Policies and the Editorial Policy Checklist.

Statistics

For all statistical analyses, confirm that the following items are present in the figure legend, table legend, main text, or Methods section.

n/a	Confirmed	
☐	☒	The exact sample size (*n*) for each experimental group/condition, given as a discrete number and unit of measurement
☐	☒	A statement on whether measurements were taken from distinct samples or whether the same sample was measured repeatedly
☐	☒	The statistical test(s) used AND whether they are one- or two-sided<br>*Only common tests should be described solely by name; describe more complex techniques in the Methods section.*
☐	☒	A description of all covariates tested
☐	☒	A description of any assumptions or corrections, such as tests of normality and adjustment for multiple comparisons
☐	☒	A full description of the statistical parameters including central tendency (e.g. means) or other basic estimates (e.g. regression coefficient) AND variation (e.g. standard deviation) or associated estimates of uncertainty (e.g. confidence intervals)
☐	☒	For null hypothesis testing, the test statistic (e.g. *F*, *t*, *r*) with confidence intervals, effect sizes, degrees of freedom and *P* value noted<br>*Give P values as exact values whenever suitable.*
☒	☐	For Bayesian analysis, information on the choice of priors and Markov chain Monte Carlo settings
☒	☐	For hierarchical and complex designs, identification of the appropriate level for tests and full reporting of outcomes
☒	☐	Estimates of effect sizes (e.g. Cohen's *d*, Pearson's *r*), indicating how they were calculated

Our web collection on statistics for biologists contains articles on many of the points above.

Software and code

Policy information about availability of computer code

Data collection	Custom software, running under Windows 10, was used to measure ocular misalignment using a perceptual-nulling technique in the vertical and torsional directions (see Ocular misalignment methods for description)<br>Butterfly Network app for iOS (v2.2.1 for pre-flight & 2.5.0 for in-flight)<br>Joggle software (v3.0.9)<br>watchOS ECG (v1.9)<br>watchOS (v7.1)
Data analysis	bbtools (v38.92)<br>XTree (v0.92i)<br>Kraken2 (v2.1.2)<br>bracken (v2.6.2)<br>vegan (v2.6.2)<br>MetaSPAdes (v3.14.3)<br>MetaQUAST (v5.0.2)<br>Bowtie2 (v2.2.3)<br>samtools (v1.0, 1.9)<br>CheckV (v0.8.1)<br>Bakta (v1.5.1)<br>MMseqs2 (v13.4511)<br>Diamond (v2.0.14)

FastQC (v0.11.9)
trimmomatic (v0.39)
Bowtie2 (v2.4.1)
bedtools (v2.29.2)
SAS (9.4)
MATLAB (2018b)
Osirix MD DICOM (v7.5)
PepSIRF (v1.4.0)
R (v4.1.2)
ImageJ (v1.52a)

For manuscripts utilizing custom algorithms or software that are central to the research but not yet described in published literature, software must be made available to editors and reviewers. We strongly encourage code deposition in a community repository (e.g. GitHub). See the Nature Portfolio guidelines for submitting code & software for further information.

Data

Policy information about availability of data

All manuscripts must include a data availability statement. This statement should provide the following information, where applicable:

- Accession codes, unique identifiers, or web links for publicly available datasets
- A description of any restrictions on data availability
- For clinical datasets or third party data, please ensure that the statement adheres to our policy

Datasets have been uploaded to three different data repositories: the NASA Open Science Data Repositories (OSDR; osdr.nasa.gov; comprised of GeneLab and the Ames Life Sciences Data Archive [ALSDA], and the TrialX database.)

Human research participants

Policy information about studies involving human research participants and Sex and Gender in Research.

Reporting on sex and gender	Yes, sex information was collected. However, due to the small sample size (N=4) and that the identity of the crew is publicly available, we do not present or analyze data for sex differences to protect the confidentiality of the Inspiration4 crew.
Population characteristics	The crew composition was of two races and ages ranged from 29-51 years.
Recruitment	Participants were recruited by SpaceX and mission commander Jared Isaacman.
Ethics oversight	All subjects were consented at an informed consent briefing (ICB) at SpaceX (Hawthorne, CA), and samples were collected and processed under the approval of the Institutional Review Board (IRB) at Weill Cornell Medicine, under Protocol # 21-05023569. All crewmembers consented to data and sample sharing.

Note that full information on the approval of the study protocol must also be provided in the manuscript.

Field-specific reporting

Please select the one below that is the best fit for your research. If you are not sure, read the appropriate sections before making your selection.

☒ Life sciences ☐ Behavioural & social sciences ☐ Ecological, evolutionary & environmental sciences

For a reference copy of the document with all sections, see nature.com/documents/nr-reporting-summary-flat.pdf

Life sciences study design

All studies must disclose on these points even when the disclosure is negative.

Sample size	The entire Inspiration4 crew was profiled, which was limited by the size of the Dragon capsule (N=4).
Data exclusions	No data were excluded.
Replication	Replication tests are difficult as mission parameters cannot be repeated. For the virome assay, assay validation and analytical performance tests were conducted in the laboratory on Earth. Furthermore, data validation was performed via western blots to validate proteomic findings.
Randomization	This is not relevant to the study as we were profiling the entire crew longitudinally (pre-flight, in-flight, and post-flight).
Blinding	Blinding was not possible because all subjects were astronauts in the same crew.

Reporting for specific materials, systems and methods

We require information from authors about some types of materials, experimental systems and methods used in many studies. Here, indicate whether each material, system or method listed is relevant to your study. If you are not sure if a list item applies to your research, read the appropriate section before selecting a response.

Materials & experimental systems

n/a	Involved in the study
☐	☒ Antibodies
☒	☐ Eukaryotic cell lines
☒	☐ Palaeontology and archaeology
☒	☐ Animals and other organisms
☐	☒ Clinical data
☒	☐ Dual use research of concern

Methods

n/a	Involved in the study
☒	☐ ChIP-seq
☒	☐ Flow cytometry
☒	☐ MRI-based neuroimaging

Antibodies

Antibodies used

Goat anti-human IgM, Fc5μ Fragment Specific (109-005-129, Jackson ImmunoResearch)
Rabbit anti-human CRP (11250-R106, SinoBiological)
Mouse anti-Human IgM (MA5-14729, ThermoFisher)
Mouse anti-human CRP (11250-MM07T, SinoBiological)
Goat anti-mouse (115-005-166, Jackson ImmunoResearch)

Validation

All capture and detection antibodies have been tested and validated to detect human recombinant protein using enzyme-linked immunosorbent assay (ELISA). In addition, rabbit and mouse anti-human C-reactive protein (CRP) was also successfully validated for ELISA application according to the manufacturer's protocol (website for rabbit: https://www.sinobiological.com/antibodies/human-c-reactive-protein-11250-r106 and mouse: https://www.sinobiological.com/antibodies/human-c-reactive-protein-11250-mm07t, respectively). Goat anti-human Immunoglobulin M (IgM) was tested for immunoassay application by the manufacturer (https://www.citeab.com/antibodies/2037259-109-005-129-affinipure-goat-anti-human-igm-fc5mu-fr?utm_campaign=Widget+All+Citations&utm_medium=Widget&utm_source=Jackson+Immunoresearch&utm_term=Jackson+ImmunoResearch), as well as by previous publications including: Gasser et al. Cell Rep. 2021 Mar 2;34(9):108790 (PMCID=PMC7874916; doi=10.1016/j.celrep.2021.108790) and Liu et al. Nat Biomed Eng. 2020 Dec;4(12):1188-1196 (doi=10.1038/s41551-020-00642-4). Mouse anti-Human IgM was also been tested and did not react with Human Immunoglobulin G (IgG) or Immunoglobulin A (IgA) via ELISA according to the manufacturer protocol (website: https://www.thermofisher.com/antibody/product/Mouse-anti-Human-IgM-Secondary-Antibody-clone-P5E2-Monoclonal/MA5-14729). In addition to our own validation studies using plasma samples, secondary goat anti-mouse did not detect non-immunoglobulin serum proteins, which was tested via ELISA and/or solid-phase adsorption using manufacturer methodology to ensure minimal cross-reaction with human serum proteins (website: https://www.jacksonimmuno.com/catalog/products/115-005-166).

Clinical data

Policy information about clinical studies
All manuscripts should comply with the ICMJE guidelines for publication of clinical research and a completed CONSORT checklist must be included with all submissions.

Clinical trial registration

The IRB was hosted at Cornell for the clinical data collection (Protocol 21-05023569), and the consent briefings were at SpaceX headquarters in Hawthorne, CA. This was not an interventional study, and thus does not require the ICMJE checklist.

Study protocol

We have included the full protocol and informed consent form (ICF) on the data portal that comes with the paper: https://soma.weill.cornell.edu.

Data collection

Recruitment was from January 2021 - April 2021, and we completed three pre-flight collections, two mid-flight collections, and three post-flight collections. Samples for Quest CBC and CMP data were shipped immediately after collection, and CLIA WGS is planned for variant interpretation and pharmoacogenomics, but not reported in this study.

Outcomes

Outcomes were quantification of the cell counts from the CBC panel and the ng/mL measurements of the metabolites. The goal was to show the post-flight measures for these cellular and metabolic counts were not significant from pre-flight levels.
